# CAMKIIδ Reinforces Lipid Metabolism and Promotes the Development of B Cell Lymphoma

**DOI:** 10.1002/advs.202409513

**Published:** 2025-01-22

**Authors:** Jiawei Zhang, Senlin Xu, Hui Fang, Dehao Wu, Ching Ouyang, Yunfei Shi, Zhenkang Hu, Mingfeng Zhang, Yaoyao Zhong, Junwei Zhao, Yichao Gan, Shize Zhang, Xiaoqian Liu, Jie Yin, Yuan Li, Mengyue Tang, Yingda Wang, Ling Li, Wing C Chan, David Horne, Mingye Feng, Wendong Huang, Ying Gu

**Affiliations:** ^1^ Cancer Institute (Key Laboratory of Cancer Prevention and Intervention China National Ministry of Education) Second Affiliated Hospital School of Medicine Zhejiang University Hangzhou 310009 China; ^2^ Molecular and Cellular Biology of Cancer Program & Department of Diabetes Complications and Metabolism Arthur Riggs Diabetes & Metabolism Research Institute Beckman Research Institute City of Hope Duarte CA 91010 USA; ^3^ Center for Genetic Medicine, the Fourth Affiliated Hospital School of Medicine Zhejiang University Yiwu 322000 China; ^4^ Institute of Genetics International School of Medicine Zhejiang University Hangzhou 310058 China; ^5^ Department of Digestive Fuzhou University Affiliated Provincial Hospital Fuzhou Fujian 350001 China; ^6^ Integrative Genomic Core City of Hope National Medical Center Duarte CA 91010 USA; ^7^ Key laboratory of Carcinogenesis and Translational Research (Ministry of Education/Beijing) Department of Pathology Peking University Cancer Hospital and Institute Beijing 100142 China; ^8^ Department of Hematology The Affiliated Yantai Yuhuangding Hospital of Qingdao University Yantai Shandong 264000 China; ^9^ Irell and Manella Graduate School of Biological Sciences Beckman Research Institute City of Hope Duarte CA 91010 USA; ^10^ Department of Hematologic Malignancies Translational Science Beckman Research Institute City of Hope National Medical Center Duarte CA 91010 USA; ^11^ Department of Pathology City of Hope National Medical Center Duarte CA 91010 USA; ^12^ Department of Molecular Medicine Beckman Research Institute City of Hope National Medical Center Duarte CA 91010 USA; ^13^ Department of Immuno‐oncology Beckman Research Institute City of Hope National Medical Center Duarte CA 91010 USA; ^14^ Zhejiang Provincial Key Lab of Genetic and Developmental Disorder Hangzhou 310058 China

**Keywords:** B cell lymphoma, CAMKIIδ, FOXO3A, lipid metabolism

## Abstract

The most prevalent types of lymphomas are B cell lymphomas (BCL). Newer therapies for BCL have improved the prognosis for many patients. However, approximately 30% with aggressive BCL either remain refractory or ultimately relapse. These patients urgently need other options. This study shows how calcium/calmodulin‐dependent protein kinase II delta (CAMKIIδ) is pivotal for BCL development. In BCL cells, ablation of CAMKIIδ inhibits both lipolysis from lipid droplets and oxidative phosphorylation (OXPHOS). With lipolysis blocked, BCL progression is markedly suppressed in two distinct BCL mouse models: MYC‐driven *EµMyc* mice and *Myc/Bcl2* double‐expressed mice. When CAMKIIδ is present, it destabilizes transcription factor Forkhead Box O3A (FOXO3A) by phosphorylating it at Ser7 and Ser12. This then permits transcription of downstream gene IRF4 – a master transcription factor of lipid metabolism. The CAMKIIδ/FOXO3A axis bolsters lipid metabolism, mitochondrial respiration, and tumor fitness in BCL under metabolic stress. This study also evaluates Tetrandrine (TET), a small molecule compound, as a potent CAMKIIδ inhibitor. TET attenuates metabolic fitness and elicits therapeutic responses both in vitro and in vivo. Collectively, this study highlights how CAMKIIδ is critical in BCL progression. The results also pave the way for innovative therapeutic strategies for treating aggressive BCL.

## Graphical abstract

Researchers have revealed a new role for the protein CAMKIIδ in promoting the development of B cell lymphomas (BCL) by boosting lipid metabolism and energy production in lymphoma cells. Furthermore, they have discovered that tetrandrine can potentially inhibit CAMKIIδ, thereby reducing the burden of lymphoma. This opens up a promising avenue for BCL treatment.

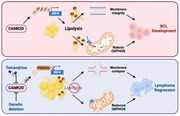

## 1 Introduction

Non‐Hodgkin lymphoma (NHL) is a common life‐threatening malignancy. In 2024, an estimated 80620 new cases of NHL will be diagnosed in the US with 20140 associated deaths.^[^ ^1^ ^]^ NHLs are classified based on the cell of origin. B‐cell lymphoma (BCL) represents the predominant subtype: it accounts for approximately 90% of cases. BCL patients are typically treated with standard chemotherapy regimens. Three recent treatment advances are targeted drugs, immunotherapy, and CAR T‐cell therapy; they have shown promising results for some relapsed and refractory cases. Still, approximately 30% of BCL patients fail to achieve a 5‐year overall survival. Particularly poor prognoses are associated with high‐grade B‐cell lymphoma (HGBL), which is characterized by MYC and BCL2 and/or BCL6 rearrangements.^[^ ^2^ ^]^ Ongoing clinical trials for BCL, especially for aggressive HGBL, require further validation. Consequently, patients urgently need new therapeutic strategies to improve BCL treatment outcomes.

Cancer cells exhibit a high energy demand. This hallmark of cancer is characterized by reprogrammed cellular metabolism; this sustains cancer cell turnover and proliferation by providing more energy fuels and building blocks. For example, one type of fuel that has been recently recognized to play a pivotal role in tumorigenesis is lipids.^[^ ^3^ ^]^ Notably, ATP generated from the oxidation of a 16‐carbon fatty acid (i.e., palmitic acid) provides three times more energy than the same amount of glucose. Meanwhile, intermediates from lipid metabolism served as building blocks for membrane synthesis and coordinated signaling transduction.^[^ ^4^ ^]^ Physiologically, either fatty acids from diets or *de novo* synthesis are bound to fatty acid‐binding proteins and delivered to the mitochondria or peroxisomes for oxidation. However, any excess fatty acids are stored in lipid droplets. These stored lipids can be broken down into free fatty acids and glycerol in a process catalyzed by lipase enzymes. The process, termed lipolysis, serves both metabolic and signaling functions. Importantly, both aberrant lipolysis and increased lipogenesis have been identified in many BCL subtypes, including mantle cell lymphoma and chronic lymphocytic leukemia.^[^ ^5^ ^]^ Elevated levels of fatty acid synthase (FASN) have been reported in double‐expressor lymphoma.^[^ ^6^ ^]^ Changes in other lipid components, such as increased cholesterol^[^ ^7^ ^]^ and cardiolipin,^[^ ^8^ ^]^ promote lymphomagenesis and are potential therapeutic targets for cancer treatment.

Ca^2+^/calmodulin‐dependent protein kinase II (CAMKII) is a multi‐functional serine/threonine kinase. CAMKII is chronically activated under pathological conditions.^[^ ^9^ ^]^ Our lab and others have extensively probed aberrant CAMKII expression and its tumor‐promoting functions in several tumors.^[^ ^10^ ^]^ Recently, CAMKII has emerged as playing a role in metabolic regulation. In the liver, CAMKII is activated in response to fasting and glucagon. Its activity enhances glucose production by promoting FOXO1 nuclear translocation.^[^ ^11^ ^]^ Glucagon‐induced CAMKII activity also stimulates lipolysis by phosphorylating ATGL (Adipose Triglyceride Lipase) and can further fuel mitochondria respiration.^[^ ^12^ ^]^ In adipose tissue, CAMKII inhibition results in reduced lipolysis.^[^ ^13^ ^]^ This evidence highlights the critical role of CAMKII in regulating both glucose and lipid metabolism.

Here, we demonstrated how CAMKIIδ, the dominant isoform of CAMKII in BCL, regulated lipid metabolism reprogramming: CAMKIIδ phosphorylated and destabilized the FOXO3A transcription factor. Inhibiting CAMKIIδ by either genetic ablation or a small molecule inhibitor suppressed lipid utilization. Moreover, its inhibition suppressed BCL development both in vitro and in vivo. Altogether, our data may provide a novel avenue for treating aggressive BCL.

## 2 Results

### 2.1 CAMK2D Is Essential for B Cell Lymphoma Development

**Figure 1:**
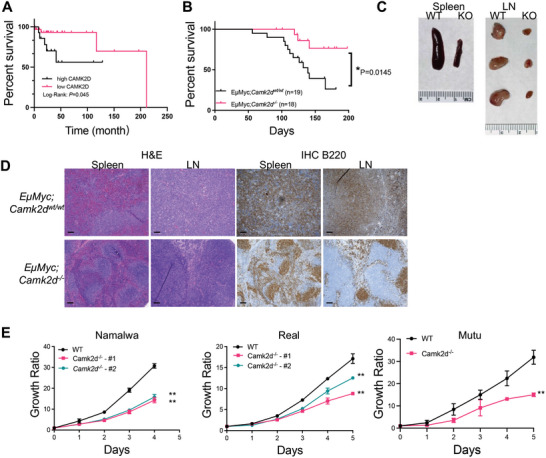
CAMKIIδ is critical for BCL proliferation. A) Kaplan–Meier survival of TCGA‐DLBCL patients with either high (n = 15) or low (n = 33) CAMK2D expression. B) Kaplan–Meier survival curves for *EµMYC; Camk2d^wt/wt^* (n = 18) and *EµMYC; Camk2d ^‐/‐^* mice (n = 19). *P* values were calculated using the log‐rank test. C) Representative anatomic images of spleens and lymph nodes from *EµMYC; Camk2d^wt/wt^* (WT) and *EµMYC; Camk2d ^‐/‐^* (KO) mice (15 weeks). D) Representative images of H&E and IHC staining for B220 (CD45R) on spleens and lymph node sections. The bar represents 100µm. E) Growth curves of indicated BCL cell lines with or without CAMK2D. Data are presented as mean ± SD from four independent biological samples for each group. All the results shown here were representative of three independent experiments. T‐test was used for analysis unless otherwise indicated.

In our previous study, we showed CAMKIIγ to be a dominant isoform of CAMKII in T cells. We also showed how CAMKIIγ stabilized c‐Myc by phosphorylating c‐Myc at Ser62. The phosphorylation subsequently led to c‐MYC oncogenic activity in T cell lymphoma (TCL).^[^ ^14^ ^]^ These prior results suggest targeting CAMKIIγ as a potential therapy for TCL with c‐Myc dysregulation. However, when we started to look at the target for other types of lymphoma, CAMKIIγ did not appear to be promising for BCL. In the TCGA‐DLBCL cohort, low CAMKIIγ expression is not correlated with a better prognosis (Figure S1A, Supporting Information). Instead, TCGA‐DLBCL samples exhibit higher CAMK2D versus CAMK2G (Figure S1B, Supporting Information). Further analysis of the TCGA‐DLBCL cohort showed that elevated CAMKIIδ expression correlates with poorer prognosis (Figure 1A). We also analyzed two additional independent DLBCL datasets,^[^ ^15^ ^]^ finding that elevated CAMKIIδ levels are associated with poorer prognosis in both (Figure S1C,D, Supporting Information). Besides, CAMKIIδ predicts poor prognosis in both ABC and GCB subtypes of DLBCL (Figure S1E, Supporting Information). Analyzing CAMKIIδ levels in lymphoma cell lines revealed CAMKIIδ as the dominant isoform in BCL (Figure S1F, Supporting Information).

The t (8;14) translocation juxtaposes the MYC gene next to the immunoglobulin locus and leads to unrestrained transcription of MYC oncogene in the B cell lineage. This translocation is prevalent in Burkitt lymphoma (BL) and a subset of aggressive diffuse large B cell lymphoma (DLBCL). The *EµMYC* mouse model mimics the MYC translocation ^[^ ^16^ ^]^ and spontaneously develops lymphoma in B cell lineage as early as 8–13 weeks. To determine the effects of CAMKIIδ, we generated whole‐body CAMK2D knockout mice *(Camk2d^‐/‐^*) and crossed them with *EµMYC* mice. Compared to the *EµMYC: Camk2d^wt/wt^* littermates, *EµMYC: Camk2d^‐/‐^* mice exhibited prolonged survival (**Figure** **1**B). Gross anatomy revealed reduced tumor infiltration in lymphoid organs, with smaller lymph nodes, and spleen in *EµMYC: Camk2d^‐/‐^* mice (Figure 1C). Histologically, *EµMYC: Camk2d^wt/wt^* mice had diffused malignant B cells (B220+) occupying most of the lymphoid tissues. In marked contrast, *EµMYC: Camk2d^‐/‐^* mice largely retained the normal lymphoid tissue architecture (Figure 1D). These results strongly suggest a link between CAMK2D and BCL lymphomagenesis. Subsequently, we knocked out the CAMK2D gene in three BL cell lines: Namalwa, Rael, and Mutu. As expected, CAMK2D deletion in all these cells significantly inhibited tumor cell proliferation (Figure 1E). Collectively, CAMKIIδ plays a pivotal role in promoting B‐cell lymphomagenesis.

### 2.2 CAMK2D Deletion Impairs Mitochondrial OXPHOS

**Figure 2:**
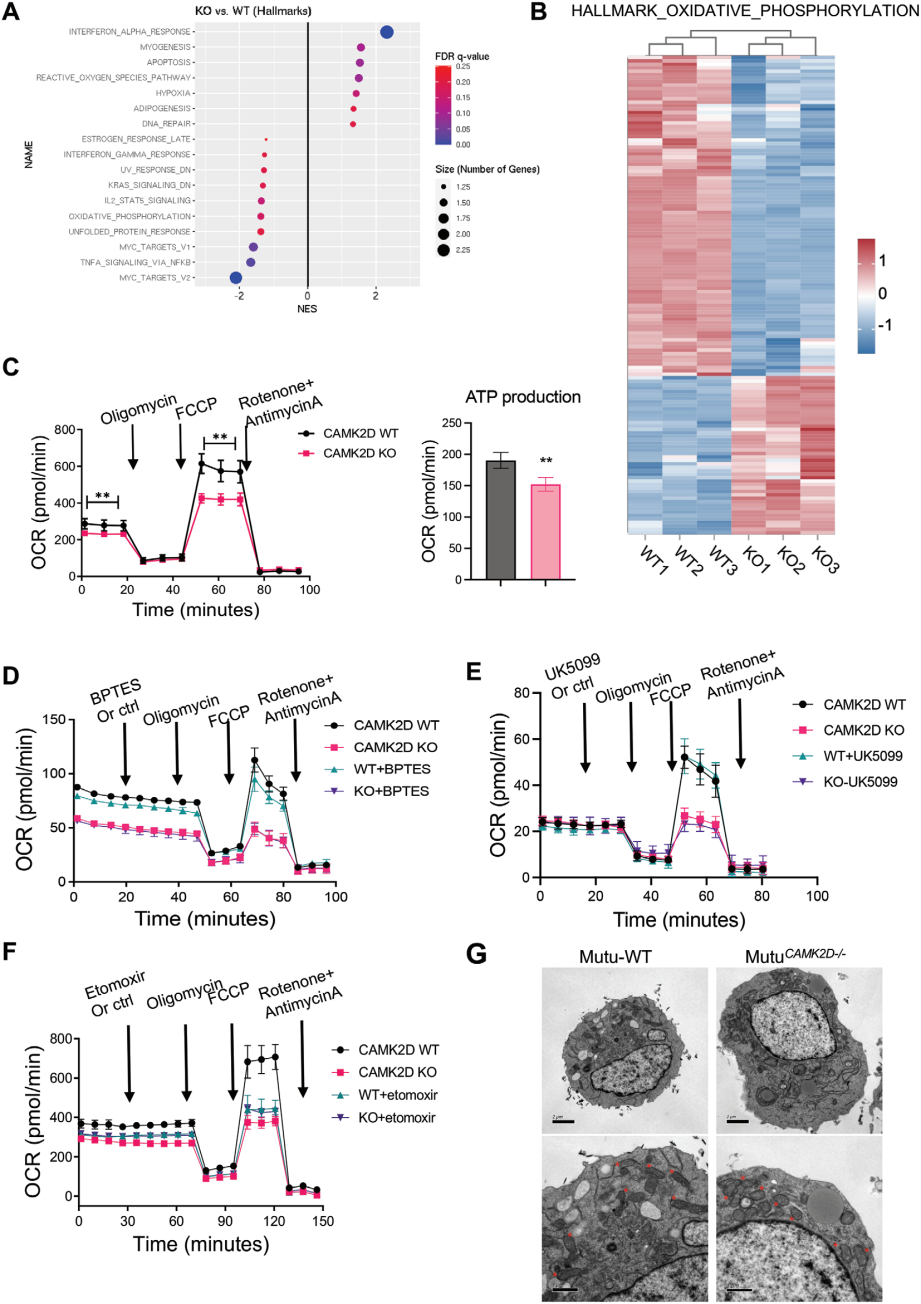
CAMK2D inhibition impairs mitochondria functions. A) GSEA analysis showed top hallmark pathways enriched in CAMK2D^‐/‐^ cells. B) Heatmap analysis of genes in OXPHOS pathways from RNA‐seq between CAMK2D WT and KO cells. C) Representative measures of oxygen consumption rate (OCR) between Mutu CAMK2D WT and KO cells upon addition of Oligomycin, FCCP, Rotenone, and AntimycinA. Quantified ATP production. **p < 0.01. Mutu CAMK2D WT and KO cells were treated with either control or BPTES (D), UK5099 (E), and etomorxir (F). Oxygen consumption rate (OCR) upon the addition of indicated inhibitors was measured by Agilent Seahorse XF Analyzers. Data in panels C‐F are presented as mean ± SD from four independent biological samples for each group. G) Representative images of the electron microscope of mitochondria in the indicated cells. The bars represent 2 µm (upper) and 1 µm (lower). The results shown in panels C‐G are representative of three independent experiments. T‐test was used for analysis, unless otherwise indicated.

To elucidate the underlying mechanisms by which CAMKIIδ promotes BCL, we performed RNA sequencing (RNA‐seq) to compare the global gene profiles between wildtype (WT) and CAMK2D^‐/‐^ BCL cells. Gene set enrichment analysis (GSEA) of hallmark pathways revealed that CAMK2D deletion altered major energy metabolism pathways: we observed 1) an increase in reactive oxygen species (ROS), hypoxia, and adipogenesis pathways; 2) a decrease in the oxidative phosphorylation (OXPHOS) pathway (**Figure** **2**A). While a panel of genes in the OXPHOS pathway are significantly downregulated after CAMK2D deletion (Figure 2B), few genes in the glycolysis pathway showed significant changes (Figure S2A, Supporting Information). Consistently, previous findings suggest CAMKIIδ plays a role in mitochondria functions and oxidative stress.^[^ ^17^ ^]^ Accordingly, Seahorse assay results showed how CAMK2D depletion resulted in decreased oxygen consumption (OCR), especially after adding uncoupling reagent FCCP. Thus, CAMK2D depletion appeared to be accompanied by a significant decrease in mitochondria respiration capacity (Figure 2C and Figure S2C, Supporting Information). In contrast, measurements of extracellular acidification rate (ECAR) suggested that CAMK2D depletion did not affect the glycolytic response and its inhibitory effect on mitochondria was not due to glycolytic flux changes (Figure S2B, Supporting Information). Reduction in OCR corresponded to decreased ATP production (Figure 2C). Inhibiting OXPHOS by Metformin also reduced B‐cell lymphoma growth (Figure S2D, Supporting Information). Collectively, the mitochondrial OXPHOS pathway appears to be critical for BCL growth.

To identify the primary substrate fueling respiration, we performed the Seahorse XF Substrate Oxidation Stress Test. Mitochondrial oxidative respiration was measured after adding one of three substrate pathway‐specific inhibitors: 1) etomoxir blocked fatty acid oxidation (FAO) of long‐chain fatty acids (LCFAs) substrates by inhibiting carnitine palmitoyl transferase 1a (CPT1a); 2) UK5099 blocked consumption of glucose and/or pyruvate by inhibiting of the mitochondrial pyruvate carrier; 3) BPTES blocked consumption of glutamine by inhibiting glutaminase 1 (GLS‐1). According to our results, impaired oxidative respiration in CAMK2D knockout cells was attributed to reduced FAO. Only the FAO inhibitor etomoxir (versus BPTES or UK5099) reduced the OCR of wildtype cells to a level comparable to CAMK2D^‐/‐^ cells (Figure 2D–F, and Figure S2E, Supporting Information). There are no general defects observed after CAMK2D knockout, as depleting glucose or glutamine from the assay medium reduced OCR equally in both wild‐type and CAMK2D^‐/‐^ cells (Figure S2F, Supporting Information). Replenishing palmitate as the source of fatty acids did not further increase the OCR levels, indicating the OCR defects of CAMK2D knockout are not due to the lack of substrates (Figure S2G, Supporting Information). It is noted that etomoxir may inhibit oxidative metabolism independent of FAO at concentrations exceeding 5µM.^[^ ^18^ ^]^ To exclude the possible off‐target effects of etomoxir, we blocked FAO using the specific FATP transporter inhibitor FATP1‐IN‐2 ^[^ ^19^ ^]^ and repeated the Seahorse assay under the same conditions as etomoxir. Consistently, the FATP1 inhibitor reduced the OCR of WT cells to a level similar to that of KO cells (Figure S2H, Supporting Information).

Impaired OXPHOS was accompanied by dysregulated mitochondria structural dynamics. Transmission electron microscopy (TEM) revealed that CAMK2D^‐/‐^ cells harbored more swollen mitochondria with fewer cristae per mitochondria. These results are consistent with functional damage of mitochondria in CAMKII‐depleted cells (Figure 2G and Figure S2I, Supporting Information).

### 2.3 CAMKIIδ Regulates Lipid Homeostasis in B‐Cell Lymphoma

Emerging research has linked altered lipid metabolism to B‐cell lymphoma. For example, one subset of DLBCL is termed OXPHOS‐DLBCL; it exhibits enhanced mitochondrial respiration,^[^ ^20^ ^]^ enhanced fatty acid oxidation, and enhanced cell proliferation fueled by exogenous fatty acids.^[^ ^21^ ^]^ Another recent study on CD37‐high DLBCL has similarly highlighted the importance of mitochondria and FAO.^[^ ^22^ ^]^ Accordingly, our results show how inhibiting FAO and lipolysis in BCL resulted in growth suppression (Figure S3A, Supporting Information); this suggests that downregulating lipid metabolism pathways could benefit BCL treatment. We thus investigated how CAMKIIδ modulates lipid metabolism pathways in BCL.

**Figure 3:**
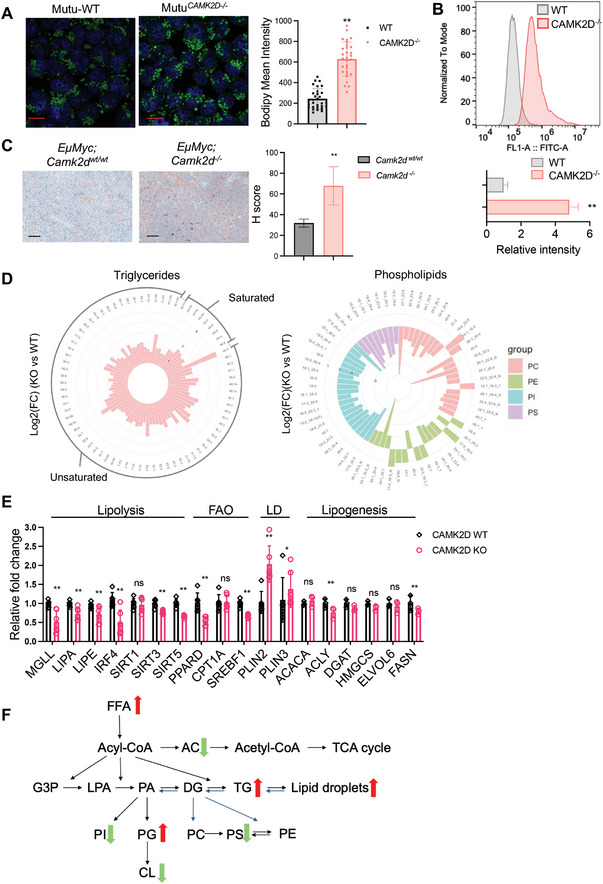
CAMKIIδ regulates lipid homeostasis in B cell lymphoma. A) Confocal microscope imaging of BODIPY 493/503 staining in cells as indicated. Blue represents DAPI staining, and green represents BODIPY staining. The bars represent 10 µm. **p < 0.01. B) Indicated cells were stained with BODIPY 493/503 and analyzed by flow cytometry. Mean fluorescent intensity (MFI) was normalized by fluorescent mode. Representative overlays between indicated cells were shown. **p < 0.01. Data are presented as mean ± SD from three technical replicates per group. C) Representative images of IHC staining for perilipin3 on *EµMYC; Camk2d^wt/wt^* and *EµMYC; Camk2d ^‐/‐^* mice spleen sections. The bars represent 50 µm. H score was determined by ImageJ. **p < 0.01. D) Lipidomic analysis was performed to compare the lipid profile of SU‐DHL‐6 CAMK2D wild‐type with knockout cells. The bar plot showed a Log2 fold change of individual (left) triglycerides, and (right) phospholipids. PC: Phosphatidylcholine; PE: Phosphatidylethanolamine; PS: Phosphatidylserine; PI: Phosphoinositide. E) mRNA expression of genes related to lipolysis, fatty acid oxidation (FAO), and lipid droplets (LD) in indicated cells. The data is representative of three experiments (average of three values ± standard error). *p < 0.05, **p < 0.01. F) Summary of the lipid components change after CAMK2D inhibition. The results shown here, except panel D, are representative of three independent experiments. T‐test was used for analysis, unless otherwise indicated.

Lipid droplets are dynamic organelles responsible for lipid storage. CAMK2D knockout cells displayed increased lipid droplet accumulation in the cytoplasm under the electron microscope (Figure 2G). Staining with the neutral lipid dye BODIPY493/503, which specifically targets cellular lipid droplets, confirmed this finding through fluorescence microscopy and flow cytometry (**Figure** **3**A,B). Consistent with this, *EµMYC: Camk2d^‐/‐^* mice exhibited increased lipid droplet accumulation in the tumor tissue, as detected by IHC with perilipin3 antibody (Figure 3C).

Lipid droplets transport a wide array of lipids with different types of structures.^[^ ^23^ ^]^ The nomenclature of these emerging metabolites is complex, and a comprehensive system for naming was recently proposed in the 2020 update on MS‐derived lipid structures (LIPID MAPS).^[^ ^24^ ^]^ Here, we focus on two components which we have termed “neutral lipids” and “phospholipids”. Examples of important neutral lipids include triacylglycerols (TG), diacylglycerols (DG), and cholesteryl esters (CE); examples of important phospholipids include phosphoinositide (PI), phosphatidylcholine (PC), and phosphatidylserine (PS). For example, PI is a minority component of cellular membranes but plays an important, instructional role by attracting specific, peripheral membrane proteins to promote normal physiological activities (e.g., membrane budding). PC is a major constituent of cell membranes and plays a role in cell signal transduction. PS is localized exclusively in the cytoplasmic leaflet of the cell membrane and is critical for protein docking and signal transduction. With this in mind, we performed lipodomic analyses focusing on these molecules to assess the impact of CAMK2D depletion. Our analyses revealed how the depletion of CAMK2D substantially increased neutral lipids TG, DG, and CE; conversely, it substantially decreased phospholipids PI, PC, and PS (Figure 3D).

Lipidomic results also revealed how CAMK2D depletion may be linked to mitochondrial dysfunction. For example, we observed marked changes to an important mitochondrial lipid called acylcarnitine (ACar) (Figure S3B), Supporting Information. ACar is converted from fatty acids by carnitine palmitoyl transferase 1 (CPT1) and shuttled into mitochondria for oxidation and energy production. CAMK2D depletion was associated with remarkably decreased ACar, reflecting impaired fatty acid oxidation. Another affected, mitochondrial lipid was cardiolipin (CL). This lipid is exclusively expressed in the mitochondrial membrane. By interacting with mitochondrial membrane proteins, CL stabilizes membrane potential and maintains the respiration chain. Importantly, CL levels were reduced in CAMK2D knockout cells. We hypothesize this reduction may be caused by impeded synthesis: the precursor to CL (Phosphatidylglycerol, (PG)) appeared to accumulate when CAMK2D was knocked out. Consistent with the previous impaired fatty acid oxidation phenotype in Figure 2F, increased levels of fatty acids (FA) were observed (Figure S3B, Supporting Information), which may damage the membrane fluidity and further suppress mitochondria functions.

According to these collective results, the depletion of CAMK2D leads to the defective breakdown of lipid droplets, significantly impairing the cells' ability to utilize them for energy. We hypothesize these lipid droplets are degraded through the lipase‐mediated pathway ^[^ ^25^ ^]^ (rather than impaired biosynthesis), which liberates fatty acids to generate energy. Specifically, gene profiles from our RNA‐seq experiments showed decreased gene expression related to lipolysis instead of biosynthesis (Figure S3C, Supporting Information). We thus compared the key enzymes and regulators in lipolysis and fatty acid oxidation pathways (Figure 3E and Figure S3D, Supporting Information). We observed decreased expression of lipase enzymes such as MGLL, HSL, and LIPA, as well as master transcriptional regulators of lipolysis like IRF4,^[^ ^26^ ^]^ SIRT3,^[^ ^27^ ^]^ and SIRT5.^[^ ^28^ ^]^ Meanwhile, lipogenesis genes and lipid transporter CD36 remained largely unchanged.

Lipotoxicity is a pathological condition characterized by excessive lipid accumulation, which can lead to excess ROS production, ER stress, and cell death. Generally, lipid droplets protect cells by sequestering excess lipids to prevent lipotoxicity.^[^ ^29^ ^]^ Dysregulation of lipid droplets, such as increased lipolysis or decreased synthesis, resulted in excess lipid accumulation. Moreover, an imbalance between saturated and unsaturated fatty acids contributes to lipotoxicity in cancer cells. ^[^ ^30^, ^31^ ^]^ Since we did not observe significant changes in the ER stress pathway after CAMK2D knockout (Figure S3E, Supporting Information) and lipidomics analysis showed alterations in both saturated and unsaturated fatty acids without significant accumulation of either, we conclude that lipotoxicity is not the primary cause of cell death in CAMK2D knockout cells.

In summary, lipid profile analysis indicated that CAMK2D knockout decreased FAO and lipolysis, which led to lipid accumulation in lymphoma cells (Figure 3F). Both limited sources of energy and compromised membrane backbone are particularly pernicious to lymphoma cell growth.

### 2.4 Identification of FOXO3A as a Key Substrate of CAMKIIδ

**Figure 4:**
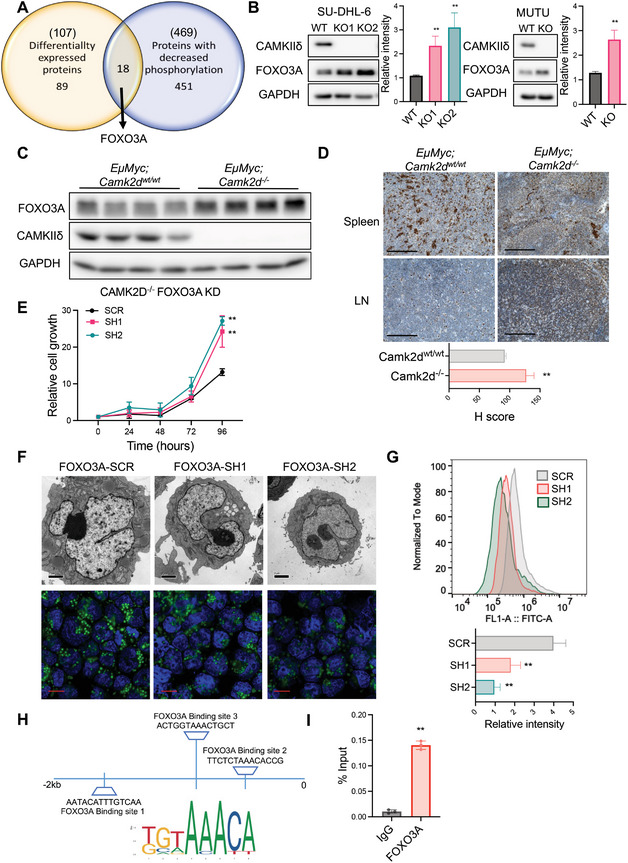
FOXO3A is a key substrate of CAMKIIδ. A) Proteomic analysis was conducted between Mutu CAMK2D wild‐type and knockout cells. The Venn diagram showed FOXO3A was one of the 18 proteins with both expression and phosphorylation changes. B) Representative western blots for FOXO3A protein levels in CAMK2D wild‐type (WT) and knockout (KO) cells. Data are presented as mean ± SD from three independent biological samples per group. **p < 0.01. C) Representative western blot and D) IHC staining of FOXO3A between *EµMYC; Camk2d ^wt/wt^* and *EµMYC; Camk2d^‐/‐^* mice. The bars represent 50µm. H score was determined by ImageJ. **p < 0.01. E) Growth curve of Mutu CAMK2D knockout cells with either FOXO3A knockdown (SH) or scramble control (SCR). Data are presented as mean ± SD from four independent biological samples per group. F) (upper) Representative images of the electron microscope of lipid droplets in the indicated cells. The bars represent 2 µm. (lower) Confocal microscope imaging of BODIPY 493/503 staining in the indicated cells. Blue represents DAPI staining, and green represents BODIPY staining. The bars represent 10 µm. G) The median fluorescent intensity (MFI) of BODIPY 493/503 in the indicated cells was analyzed by flow cytometry. MFI was normalized by fluorescent mode. Representative overlays among the indicated cells were shown. Data are presented as mean ± SD from three technical replicates. H) Diagram of FOXO3A binding on IRF4 promoter region. I) The enrichment of FOXO3A on the IRF4 promoter region was examined by ChIP. The enriched DNA sequence was detected by qPCR. **p < 0.01 in FOXO3A‐ChIP compared to IgG. All the results shown here, except panel A, are representative of three independent experiments. T‐test was used for analysis, unless otherwise indicated.

Because CAMKIIδ is a serine/threonine kinase, we postulated that its substrates regulate the transcription of lipid metabolic genes in lymphoma cells. We thus conducted proteomic analysis between Mutu CAMK2D WT and knockout cells. CAMK2D knockout significantly changed the expression levels of 107 proteins. Additionally, the knockout produced changes in phosphorylation at specific amino acid sites in 469 proteins. Overlapping the 107 with the 469 proteins resulted in 18 potential CAMKIIδ phosphorylation substrates (**Figure** **4**A, and **Table** **1**). We further validated protein abundance using Western Blot analyses (Figure S4A, Supporting Information). While other top candidates such as PHF23, SLC43A1, and PERK did not show consistent changes, the transcription factor FOXO3A emerged as a strong candidate for mediating the effects of CAMKIIδ on lipid metabolism and lymphomagenesis. Considered a tumor suppressor, FOXO3A can regulate c‐Myc protein levels.^[^ ^32^ ^]^ Deletion of FOXO3A accelerates lymphoma development in *EµMYC* mice.^[^ ^33^ ^]^ In the clinic, higher FOXO3A levels predict better prognosis in colorectal cancer.^[^ ^34^ ^]^ Additionally, elevated FOXO3A levels correlate with improved overall survival in the public DLBCL dataset ^[^ ^15b^ ^]^ (Figure S4B, Supporting Information). Interestingly, CAMK2D depletion did not affect the gene expression of FOXO3A, which suggests post‐transcriptional regulation of FOXO3A by CAMKIIδ. Western blot analysis confirmed increased FOXO3A protein levels in CAMK2D^‐/‐^ BCL cells (Figure 4B), and higher FOXO3A expression in *EµMYC*; *Camk2d^‐/‐^* mice compared to the wild‐type littermates (Figure 4C,D, and Figure S4C, Supporting Information).

**Table 1:** CAMKIIδ phosphorylation substrates.

Gene name	KO/WT Ratio	Regulated Type
PHF23	6.843	Up
SLC43A1	3.662	Up
FBXO46	2.824	Up
FOXO3	2.663	Up
SNX15	2.279	Up
EIF2AK3	2.14	Up
RAD51AP1	2.106	Up
RC3H1	1.853	Up
STX16	1.762	Up
DNAJC1	1.743	Up
CNOT4	1.725	Up
SNAPC4	1.677	Up
ABLIM1	1.656	Up
RASSF6	1.635	Up
ATG16L1	1.581	Up
AAGAB	1.574	Up
LENG1	0.664	Down
SLC7A6OS	0.653	Down

FOXO3A can regulate lipid metabolism and alter lipid droplets.^[^ ^35^ ^]^ We first assessed the impact of FOXO3A on mitochondrial OXPHOS and lipid metabolism in our B cell lymphoma system. Overexpressed FOXO3A in wild‐type cells (Figure S4D, Supporting Information) led to increased lipid droplet accumulation and decreased OCR (Figure S4E,F, Supporting Information). To validate FOXO3A as a downstream mediator of CAMKIIδ‐induced tumor growth, we used shRNA to knock down FOXO3A in Mutu CAMK2D knockout cells (Figure 4D). As expected, FOXO3A knockdown restored lymphoma cell growth (Figure 4E). Moreover, FOXO3A knockdown restored mitochondrial morphology, decreased the accumulation of lipid droplets in knockout cells, and enhanced mitochondrial OXPHOS (Figure 4F,G; Figure S4F, Supporting Information).

As a transcription factor, FOXO3A regulates the transcription of downstream genes by binding to specific promoter regions. We used JASPAR to predict its binding to each of the differentially expressed genes from Figure 3E. The gene target with the highest score was IRF4. This transcription factor plays an extensive role in energy metabolism.^[^ ^26^, ^36^ ^]^ It can be transcriptionally regulated by FOXO1, which belongs to the FOXO family together with FOXO3A, and boosts lipolysis by directly promoting downstream lipase gene expression.^[^ ^26^ ^]^ According to our RNA‐seq results, IRF4 is the top differentially expressed gene after CAMK2D knockout (Figure S4G, Supporting Information). Moreover, the knockdown of FOXO3A restored IRF4 expression, which suggests that FOXO3A directly suppresses IRF4 expression (Figure S4H, Supporting Information). We thus performed FOXO3A ChIP‐qPCR for IRF4 (Figure 4H and Figure S4I, Supporting Information). As shown in Figure 4I, a strong enrichment of FOXO3A binding on the IRF4 promoter region was detected, and CAMK2D knockout significantly increased the binding, resulting in enhanced suppression as detected.

Altogether, FOXO3A appears to be critical for CAMK2D‐dependent rewiring of lipid metabolism in BCL.

### 2.5 CAMKIIδ Phosphorylates FOXO3A and Decreases Its Stability

**Figure 5:**
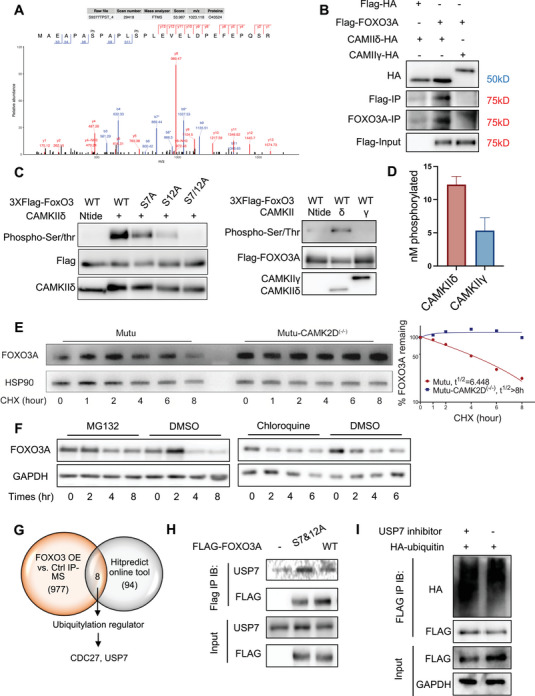
CAMKIIδ phosphorylates and destabilizes FOXO3A. A) Identification of FOXO3A S7 and S12 phosphorylation by CAMKIIδ from proteomic analysis. The mass shift of phosphorylation is 79.9663. Determination of S7: The theoretical molecular weight of SPA's three amino acid residues is 255.12. The calculation (b9−b6 = 1135.61−800.4249 = 355.185) matches the theoretical shift for SPA plus phosphorylation, calculated as (255.12+79.9663 = 355.086). Thus, it can be determined that the serine residue in SPA underwent phosphorylation modification. Determination of S12: The theoretical molecular weight of the peptide plus the charge is 2909.4. The actual mass shift across the entire peptide was calculated as (m/z × charge = 1023.118 × 3 = 3069.354), suggesting there are two phosphorylation sites on this peptide (2909.4+79.9663 × 2 = 3069.33). Furthermore, the y2−y1 fragment analysis indicates that S26 did not undergo phosphorylation modification. Since S7 was confirmed to be phosphorylated, it can be inferred that S12 is another phosphorylation site. B) HEK293 cells transfected with 3xFLAG‐FOXO3A, and CAMK2D‐HA or CAMK2G‐HA plasmids were co‐immunoprecipitated with HA‐beads; protein levels of 3xFLAG‐FOXO3A were analyzed by western blot. C) (left) In vitro kinase assay of CAMKIIδ on FOXO3A wild‐type and site mutants. HEK293 cells were transfected with 3xFLAG‐FOXO3A wild‐type, 3xFLAG‐FOXO3A S7A, S12A, and S7/12A plasmids. Proteins were then pulled down by FLAG beads, followed by an in vitro kinase assay supplied with Ca^2+^, calmodulin, and ATP. Total phosphorylated serine and threonine levels were detected by western blots. (right) In vitro kinase assay of CAMKIIδ and CAMKIIγ on FOXO3A protein. D) Radioisotope‐based in vitro kinase assay was performed on purified FOXO3A protein using 4 nM CAMKIIγ and 0.1 nM CAMKIIδ. E) Mutu CAMK2D wild‐type and knockout cells were treated with cycloheximide (CHX) for the indicated times. FOXO3A protein levels were then analyzed by western blot with Hsp90 as the loading control (left panel). Protein levels were measured with densitometric intensity. FOXO3A levels were quantified relative to Hsp90 levels and graphed as the percentage of remaining FOXO3A protein after treatment (right panel). F) Wild‐type BCL cells were treated with MG132, chloroquine, or DMSO control for indicated times, and FOXO3A expression was determined by western blot. GAPDH was used as a loading control. G) Venn diagram showing identified potential ubiquitylation regulator for FOXO3A by overlapping the MS results and online prediction. H) HEK293 cells transfected with vector control, WT or S7&12 mutant 3xFLAG‐FOXO3A were co‐immunoprecipitated with FLAG‐beads; protein levels of USP7 were analyzed by western blot. I) HEK293 cells overexpressing FLAG‐FOXO3A were transfected with HA‐ubiquitin and treated with 0.5µM USP7 inhibitor for 24 h. FOXO3A immunoprecipitated with FLAG beads, and HA‐ubiquitin was examined by western blot. All the results shown here, except panel A and G, are representative of three independent experiments. T‐test was used for analysis, unless otherwise indicated.

We performed proteomic analysis to probe the phosphorylation status of FOXO3A after CAMK2D depletion. In CAMK2D^‐/‐^ cells, FOXO3A phosphorylation was significantly reduced on Ser7 and Ser12 (**Figure** **5**A). To determine whether CAMKIIδ can phosphorylate FOXO3A and regulate FOXO3A protein degradation, we performed the following three assays. 1) We co‐transfected FOXO3A and CAMKII into HEK293T cells and conducted co‐immunoprecipitation. According to our results, FOXO3A physically interacted with and was pulled down by CAMKIIδ (Figure 5B). This interaction is specific to CAMKIIδ, as CAMKIIγ did not pull down FOXO3A. The interaction between CAMKIIδ and FOXO3A was also observed in B cell lymphoma cell lines (Figure S5A, Supporting Information). 2) We performed in vitro kinase assays. Wild‐type FOXO3A was phosphorylated in the presence of CAMKIIδ but not CAMKIIγ; phosphorylation levels were significantly decreased when Ser7 and Ser12 were mutated (Figure 5C). 3) We analyzed phosphorylation levels in vitro kinase using more sensitive radioisotope‐based assays. Although the amount of CAMKIIγ (4nM) was 40 times greater than CAMKIIδ (0.1 nM), FOXO3A phosphorylation was still higher in the CAMKIIδ kinase group (Figure 5D).

We then assessed whether the stability of FOXO3A was reduced when it was phosphorylated by CAMKIIδ. We treated both CAMK2D wild‐type and knockout cells with cycloheximide, a translation inhibitor, and measured the remaining FOXO3A protein after different time intervals. In CAMK2D^‐/‐^ cells, FOXO3A exhibited slower degradation and prolonged stability (Figure 5E). When CAMKIIδ was available in WT cells, the resulting phosphorylation events appeared to destabilize the FOXO3A protein. To further correlate FOXO3A phosphorylation sites with its protein stability, we first overexpressed either wild‐type or S7&12 mutant FOXO3A in HEK 293T cells. Then, we treated it with cycloheximide and assessed protein degradation. In S7&12 mutants, we observed a longer FOXO3A half‐life (Figure S5B, Supporting Information). We further overexpressed CAMKIIδ with wild‐type or mutant FOXO3A. Higher CAMKIIδ levels resulted in a shorter half‐life of WT FOXO3A but did not affect mutant FOXO3A (Figure S5C, Supporting Information).

To understand the mechanisms by which phosphorylation affects FOXO3A protein degradation, we performed the following experiments: first, we determined the dominant degradation pathway for FOXO3A in BCL. Proteins are generally degraded through the ubiquitin‐proteasome system (UPS) or lysosomal pathway. We treated wild‐type BCL cells with cycloheximide (CHX) and added either the proteasome inhibitor MG132 or autophagy inhibitor Chloroquine. MG132, but not Chloroquine, significantly inhibited protein degradation, suggesting that FOXO3A is degraded through the proteasomal pathway in BCL (Figure 5F). Next, we identified the critical mediator responsible for the proteasomal degradation of phosphorylated FOXO3A. HEK293 cells overexpressing FLAG‐FOXO3A were subjected to co‐IP assay with FLAG beads, and the IP complex was analyzed by mass spectrometry. This analysis identified 711 potential proteins that bind to FOXO3A. Using the online protein‐protein interactions tool (https://www.hitpredict.org/index.html), we narrowed down the candidates to CDC27 and USP7 (Figure 5G). The co‐immunoprecipitation assay confirmed the binding of FOXO3A to USP7 but not to CDC27 (Figure 5H and Figure S5D, Supporting Information). USP7 is a deubiquitinating enzyme previously reported to interact with FOXO3A.^[^ ^37^ ^]^ Moreover, the S7 and 12 mutant FOXO3A showed a higher binding affinity for USP7. Inhibiting the USP7 with its inhibitor increased the ubiquitination of FOXO3A (Figure 5I) and significantly decreased FOXO3A protein abundance (Figure S5E, Supporting Information).

Collectively, we demonstrated that CAMKIIδ phosphorylated FOXO3A specifically at Serine 7 and 12 sites, thus decreasing its binding with USP7 and promoting its degradation through the ubiquitin‐proteasome system.

### 2.6 CAMKIIδ Inhibition Halted Aggressive DHL Lymphoma

Considering that double‐hit lymphoma (DHL) is clinically more aggressive and lacks effective treatments, we investigated whether CAMKIIδ could serve as a therapeutic target for DHL. To recapitulate the DHL, we generated the MYC and BCL‐2 double‐expressed (DEL) mouse model (Figure S6A, Supporting Information). MYC and BCL‐2 conditional knock‐in mice were crossed with CD19‐Cre mice to achieve MYC and BCL‐2 overexpression specifically in the B cell lineage. The overexpressed MYC and BCL‐2 were confirmed by western blot (Figure S6B, Supporting Information). The DEL strain was highly tumorigenic, developing lymphoma with visible signs as early as 10 weeks. Mice typically succumbed to the disease at around 17 weeks or were euthanized when they became severely ill. Autopsy of the dead mice all manifested with enlargement of lymphoid organs and splenomegaly (Figure S6C, Supporting Information), with histological analysis showing densely packed malignant B cells and disrupted tissue architecture (Figure S6D, Supporting Information).

**Figure 6:**
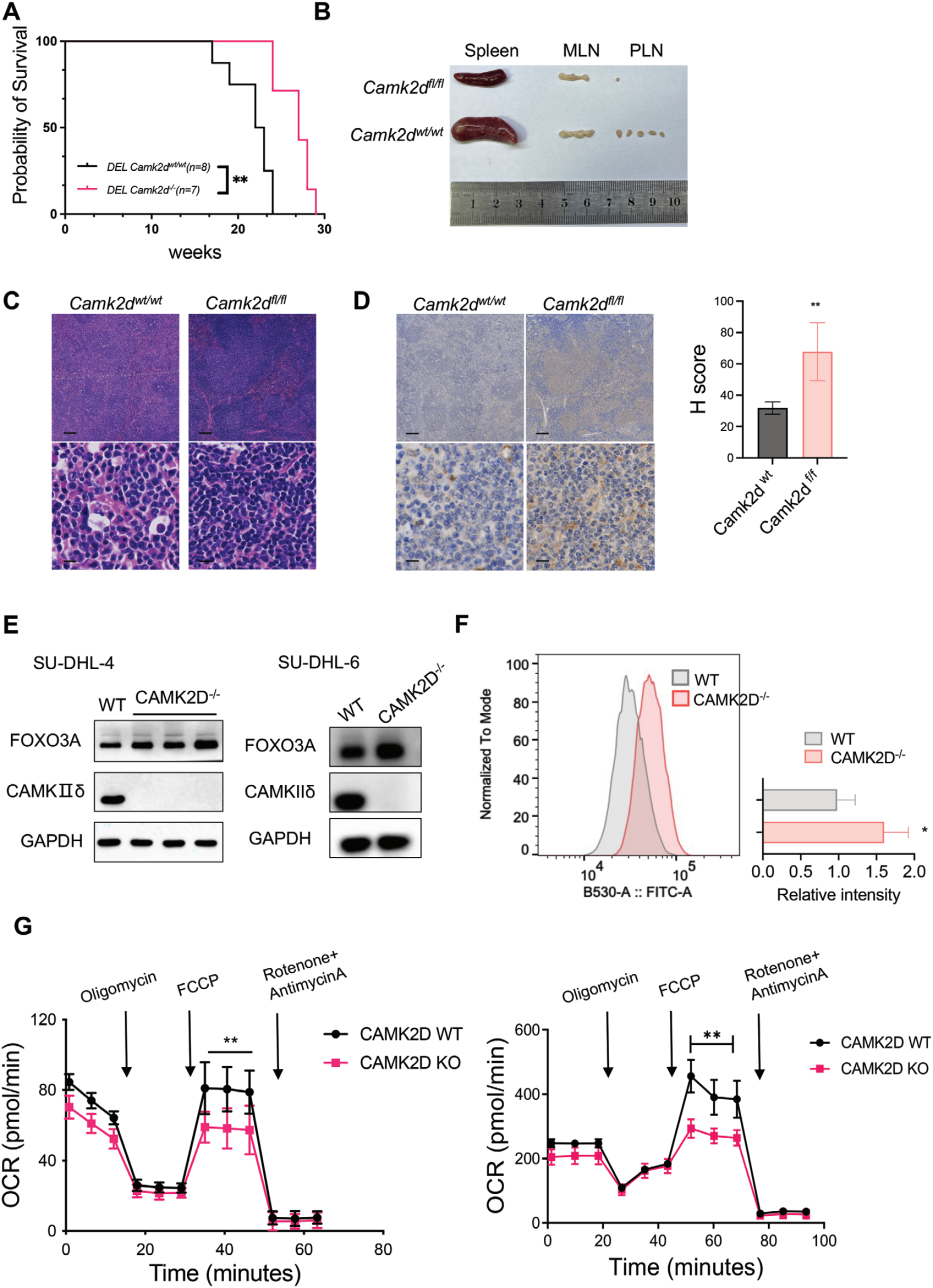
CAMKIIδ deficiency suppresses DEL development. A) Kaplan–Meier survival curves for *DEL; Camk2d^wt/wt^* (n = 8) and *DEL; Camk2d ^‐/‐^* mice (n = 7). P‐values were calculated using the log‐rank test. **p < 0.01. B) Representative anatomic images of spleens and lymph nodes from *DEL; Camk2d^wt/wt^* and *DEL; Camk2d ^‐/‐^* mice (15 weeks). Representative images of H&E C) and IHC staining for plin3 D) on spleen tissue section. The bars represent 200 µm (upper) and 10 µm (lower magnified). H score was determined by ImageJ. **p < 0.01. E) CAMK2D was knockout in two DHL cell lines. Indicated protein expression levels were detected by western blot. F) A representative overlay of the fluorescent intensity of BODIPY 493/503 in the indicated cells was analyzed by flow cytometry. **p < 0.01. Data are presented as mean ± SD from three technical replicates. G) Representative measures of oxygen consumption rate (OCR) between CAMK2D WT and KO cells. Data are presented as mean ± SD from four independent biological samples per group. **p < 0.01. T‐test was used for analysis, unless otherwise indicated. All the results shown here are representative of three independent experiments.

We then generated *Camk2d^flox/flox^*, *Myc and Bcl2^ki/wt^, CD19cre^±^* (*Camk2d^‐/‐^ DEL*), and the control littermate *Camk2d^wt/wt^*, *Myc and Bcl2^ki/wt^, CD19cre^±^* (*Camk2d^wt/wt^ DEL*) mice (Figure S6E, Supporting Information). Knockout CAMK2D in B‐cell lineage also suppressed DEL lymphoma growth: we observed delayed onset of lymphoma and death, reduced lymph node enlargement, and splenomegaly (**Figure** **6**A,B). Moreover, *Camk2d^‐/‐^ DEL* mice had less infiltration of tumors compared with *Camk2d^wt/wt^ DEL* mice (Figure 6C). These mice appeared to have dysregulated lipid metabolism: compared to the wild type, they had more lipid droplets (Figure 6D). We also generated CAMK2D knockout in DHL cell lines (Figure 6E). Consistent with previous BL cell lines, CAMK2D knockout elevated FOXO3A expression, increased the accumulation of lipid droplets, and impaired mitochondria respiration (Figure 6F and G).

### 2.7 Tetrandrine Directly Inhibits CAMKIIδ Kinase Activity

**Figure 7:**
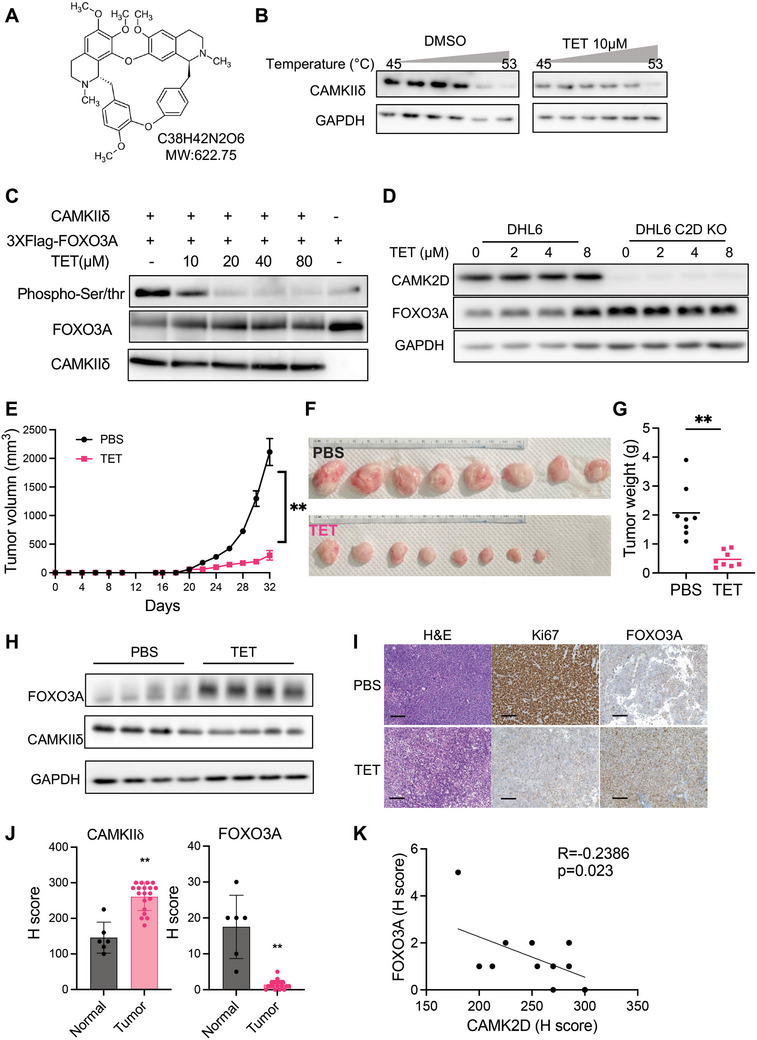
TET inhibits CAMKIIδ activity and B cell lymphoma proliferation in vitro and in vivo. A) Molecular structure of Tetrandrine (TET). B) CETSA assay to determine the physical interaction between TET and CAMKIIδ. Cells were incubated with either DMSO or 10 µM TET for 1 h and cell lysates were incubated with gradient temperature from 45 °C to 52.5 °C. CAMKIIδ protein levels were detected by western blot assay. C) In vitro kinase assay of CAMKIIδ on FOXO3A in present with TET. HEK293 cells were transfected with 3xFLAG‐FOXO3A plasmid and FOXO3A protein was pulled down by FLAG‐beads. In vitro kinase assay was performed with purified CAMKIIδ protein on FLAG‐tagged FOXO3A, incubated with calcium, calmodulin, ATP, and indicated concentrations of TET. Total phosphorylated serine and threonine levels were detected by western blotting. D) Representative western blot of SU‐DHL‐6 CAMK2D WT and KO cells treated with indicated dosages of TET for 24 h. Tumor volume growth curve E) and tumor gross pictures F) of NSG mice treated with either PBS or TET. G) Tumor weight at the endpoint of the 32nd day. **p < 0.01 versus PBS group. H) Representative western blot analysis of FOXO3A and CAMKIIδ protein levels in tumor tissues from PBS and TET treated groups. GAPDH is used as the loading control. I) Representative images of H&E and IHC staining of Ki67 and FOXO3A with sections of tumors from PBS and TET‐treated groups. The bar represents 50µm. J) IHC scores for CAMKIIδ and FOXO3A. K) Correlation of overall CAMKIIδ and nuclear FOXO3A levels. The P‐value was evaluated by Pearson Correlation assay. T‐test was used for analysis unless otherwise indicated. All the results shown here are representative of three independent experiments.

We next probed CAMKIIδ inhibition using the natural compound Tetrandrine (TET) (**Figure** **7**A) in our BCL models. TET has been reported to target CAMKIIδ, and computer modeling by the All‐Around Docking (AAD) method has shown how TET may bind to CAMKIIδ and calmodulin (CaM) complex.^[^ ^38^ ^]^ To verify TET's binding to CAMKIIδ at the cellular level, we conducted cellular thermal shift assays (CETSA) ^[^ ^39^ ^]^ in Mutu cells. In this assay, unbounded proteins are denatured and precipitated at elevated temperatures, while drug‐bound proteins remain in solution. As shown in Figure 7B, CAMKIIδ began to degrade at 50 °C in the DMSO‐treated group. However, incubation of cells with 10 µM TET for 1 h increased the degradation temperature to 53 °C, indicating TET's binding affinity to CAMKIIδ. Owing to the unavailability of commercial antibodies specific to the phosphorylated serine residues at positions 7 and 12 on FOXO3A, the Flag‐FOXO3A fusion protein was transiently overexpressed in 293T cells and subsequently purified using anti‐Flag magnetic beads. The phosphorylation status of the purified Flag‐FOXO3A protein was assessed utilizing a pan‐phosphorylated Ser/Thr antibody. Results indicated that treatment with TET markedly diminished the phosphorylation levels of the Flag‐FOXO3A protein in 293T cells (Figure 7C). We then treated multiple BCL cell lines with TET, which demonstrated strong growth inhibition with an IC_50_ value of approximately 5 µM (Figure S7A, Supporting Information). The TET treatment did not appear to inhibit the growth of CAMK2D knockout cells as much as wild‐type cells (Figure S7B, Supporting Information). Accordingly, TET treatment increased the abundance of FOXO3A protein in wild‐type cells but not in CAMK2D knockout cells (Figure 7D). Consistent with CAMK2D knockout phenotypes, TET treatment also reduced the cellular OXPHOS (Figure S7C, Supporting Information).

### 2.8 TET Inhibits B Cell Lymphoma Growth In Vivo

To determine the in vivo efficacy of TET, we used a xenografted mouse model of BCL. 5 × 10^5^ Mutu cells were inoculated into the flank of NOD. Cg‐*Prkdc^scid^ Il2rg^1Wjl^*/SzJ (NSG) mice. Once tumors reached a volume of 30 mm^3^ (day 20 post‐injection), mice were randomized into two treatment groups: phosphate‐buffered saline (PBS, control) and 100 mg kg^−1^ TET treatment (Figure S7D Supporting Information). All treatments were administered via oral gavage daily for 12 days. Compared to the PBS group, treatment with TET significantly reduced tumor volume (Figures 7E–G, and Figure S7E Supporting Information). Additionally, TET treatment did not affect body weight in either group (Figure S7F Supporting Information). On day 32, when all mice were euthanized, proteins were extracted from the tumor tissues for immunoblot to measure CAMKIIδ and FOXO3A levels (Figure 7H). Tumor tissues were processed for H&E staining and immunostained for both Ki67 and FOXO3A. Compared to the xenografted tumors from the PBS group, those from the TET group had lower levels of Ki67 and increased levels of FOXO3A (Figure 7I and Figure S7G Supporting Information).

We also assessed the efficacy of TET on wild‐type *DEL* mice. Histological analysis showed that TET treatment decreased the expression of CAMK2D and Ki67 while increasing the expression of FOXO3A (Figure S7H Supporting Information). These results demonstrated that TET effectively suppressed lymphoma proliferation in vivo by inhibiting the CAMKIIδ/FOXO3A axis.

### 2.9 The Protein Levels of CAMKIIδ and FOXO3A Are Negatively Correlated in Human B Cell Lymphoma

We next investigate the potential correlation between CAMKIIδ and FOXO3A protein in human DLBCL tissue. Protein levels were measured in specimens from 19 DLBCL patients, including both GCB and non‐GCB subtypes. CAMKIIδ was frequently and highly expressed (IHC score≥100) in DLBCL cases, with all positive cases exhibiting a cytoplasmic expression pattern. Compared to the benign lymph node tissues, CAMKIIδ expression was significantly higher in the DLBCL samples (Figure 7J, and Figure S7I Supporting Information). Conversely, FOXO3A exhibited a nuclear expression pattern and was significantly lower in DLBCL compared to that in benign lymph node tissues (Figure S7I Supporting Information). More importantly, there was a negative correlation between CAMKIIδ and FOXO3A expression (R = ‐0.2386, p = 0.023) (Figure 7K). This result is consistent with an inhibitory effect of CAMKIIδ on FOXO3A protein abundance.

## 3 Discussion

Herein, we systematically mapped the roles of CAMKIIδ and its underlying mechanisms in B cell lymphomagenesis. We also identified a novel CAMKIIδ/FOXO3A axis that regulates lipid metabolism in B cell lymphoma. Specifically, CAMKIIδ appeared to phosphorylate FOXO3A at Serine 7 and 12. These phosphorylation events destabilized it and reduced FOXO3A protein levels in BCL. FOXO3A is an important transcription factor that controls gene expression involved in metabolism, cancer, and lifespan.^[^ ^40^ ^]^ In our experiments, decreased levels of FOXO3A in BCL up‐regulated expression of genes in fatty acid oxidation and lipolysis – both fueled the growth of malignant B cells. Inhibition of CAMKIIδ by multiple approaches appeared to halt the lipid turnover and shut down the energy supply. We then observed tumor regression in BCL.

The roles CAMKII kinases play in cardiovascular diseases and neuron development have been extensively studied. However, fewer studies have focused on their roles in cancer development and energy metabolism. Throughout years of work from our lab and other groups, we have shed light on the cancer‐promotive roles of CAMKII in different cancers. Increased CAMKII has been observed in liver cancer,^[^ ^10^ ^b]^ colon cancer,^[^ ^41^ ^]^ breast cancer,^[^ ^42^ ^]^ lung cancer,^[^ ^43^ ^]^ and hematopoietic cancers.^[^ ^10^, ^14^, ^44^ ^]^ In general, the mechanisms by which CAMKII promotes cancers from previous studies are limited to the regulation of signaling pathways directly related to tumor growth such as c‐Myc, JAK/STAT, and AKT pathways. Here, we have shifted our focus to how CAMKII affects lipid metabolism in BCL. This emerging and important type of fuel has been recognized to play a pivotal role in tumorigenesis.

CAMKII has been shown to regulate lipid metabolism in normal adipocytes and hepatocytes.^[^ ^12^, ^45^ ^]^ However, no research has been conducted on how CAMKII may regulate lipids in cancer cells. To the best of our knowledge, this is the first time CAMKIIδ has been shown to promote lymphomagenesis by (indirectly) regulating lipid metabolism in BCL. This data may be broadly applicable. It may help to clarify emerging links between obesity (with dysregulated lipid metabolism) and the increased risk of other types of cancers: colon cancer, breast cancer, and liver cancer.

In addition to altering major metabolic pathways, our gene set enrichment analysis (Figure 2A) showed that other cancer‐related pathways, including the general apoptosis pathway and inflammation pathways such as INFα response, TNFα response, and INFγ response, are affected after CAMK2D knockout. Although inflammation pathways are potential targets for cancer treatment, they are not the direct cause of tumorigenesis. Metabolism dysregulation may be the initial step, with inflammation factors such as TNFα acting as secondary responders mediating the metabolic effects.^[^ ^46^ ^]^ Given the roles of CAMK2D in metabolism mentioned above, we considered OXPHOS to be the most dominant mechanism. Additionally, the potential role of CAMK2D in immunity requires further investigation.

We also identified FOXO3A as an important downstream target of CAMKIIδ for indirectly mediating lipid metabolism: CAMKIIδ phosphorylated FOXO3A at S7 and S12, which reduced the levels of FOXO3A. We thus have hypothesized that depletion of CAMK2D increases FOXO3A, which impairs lipid turnover and BCL growth. However, we acknowledge at least two other mechanisms may be in play. 1) CAMKII can regulate lipolysis in the liver directly by affecting levels of the liver enzyme ATGL.^[^ ^12^ ^]^ In our particular proteomic analysis, we did not observe significant changes in protein abundance or phosphorylation levels of such lipid enzymes. In the future, we plan to probe the impact of CAMK2D depletion on other direct modes of regulating lipid metabolism. 2) FOXO3A can be phosphorylated by PI3K/Akt at T32, S253, and S315. Phosphorylation at T32 and S253 creates binding sites for the chaperone protein 14‐3‐3, which sequestrates FOXO3A in the cytoplasm and prevents its transcription function.^[^ ^47^ ^]^ In our hands, we did not observe an alteration of the PI3K/Akt pathway. The PI3K/Akt pathway has been reported as one of the downstream targets of MYC‐dependent metabolic reprogramming in MYC‐driven BCL.^[^ ^48^ ^]^ Our previous work demonstrated that inhibiting CAMKIIγ downregulated c‐Myc levels.^[^ ^14^, ^49^ ^]^ However, we did not observe an alteration of the PI3K/Akt pathway along with c‐Myc inhibition.^[^ ^14^ ^]^ Therefore, regulation of FOXO3A by CAMKIIδ is independent of c‐Myc regulation in BCL. Thus, c‐Myc and FOXO3A may be two independent CAMKII downstream substrates, which work together to regulate lymphoma cell metabolism and growth.

MYC is an oncogene that regulates cellular metabolism and tumorigenesis.^[^ ^50^ ^]^ While c‐Myc regulation in glycolysis and glutamate metabolism has been extensively studied, its roles in lipid metabolism are not well known. In general, c‐Myc is considered to promote lipogenesis by activating the expression of genes in fatty acid synthesis such as ACLY, FASN, ACACA, etc.^[^ ^51^ ^]^ Lipid metabolism alterations may be also secondary to other MYC‐induced metabolic changes such as mitochondrial dysfunction.^[^ ^52^ ^]^ In our BCL models, we did not observe significant expression changes in lipogenesis genes such as ACACA, FASN, DGAT, etc.

The full enzyme of CAMKII is composed of 12 subunits. Each subunit may be one of four distinct isoforms: CAMKIIα, CAMKIIβ, CAMKIIδ, and CAMKIIγ. Although structurally similar, each isoform functions differently. They have different sub‐cellular localization as well as kinetics for calcium and other protein binding.^[^ ^53^ ^]^ While CAMKIIα and CAMKIIβ are hardly detected in lymphoid tissues, expression of CAMKIIδ and CAMKIIγ are abundant. Interestingly, we demonstrated how the CAMKIIδ/FOXO3A axis in BCL involves the CAMKIIδ isoform since only CAMKIIδ is capable of phosphorylating FOXO3A. We thus hypothesize that lipid‐related phenotypes may be more specific to CAMKIIδ. Previous studies on CAMKIIs also suggested distinct roles of CAMKII isoforms in different tissues. CAMKIIγ is required for T cell memory and development.^[^ ^54^ ^]^ CAMKIIδ is necessary for Ca^2+^ homeostasis in hearts and regulates cardiomyocyte metabolism, thus regulating cardiac function.^[^ ^55^ ^]^ Besides regulating metabolism in the heart, there is also evidence that CAMKIIδ, but not other isoforms, phosphorylates CEACAM1 specifically,^[^ ^56^ ^]^ which regulates lipids storage in hepatocytes.^[^ ^57^ ^]^ This also suggested that future studies on the CAMKII family should distinguish each isoform in different tissues. Whether other CAMKII isoforms also regulate lipid metabolism in cancers remains unknown.

Lipid droplets have been implicated in cancers.^[^ ^58^ ^]^ In lymphoma, increased adipophilin has been correlated with Burkitt lymphoma but not DLBCL.^[^ ^59^ ^]^ The role of lipid droplets in cancer development remains controversial; it is unclear whether they are a cause or a consequence. Evidence suggests that lipid droplets detoxify excess lipids, making their turnover crucial.^[^ ^60^ ^]^ In this study, we demonstrated that tumor suppression‐associated lipid droplet accumulation is the result of defective lipid utilization. The accumulation of lipid droplets due to decreased turnover and fatty acid oxidation, which suppresses tumor growth, has also been observed in AML.^[^ ^61^ ^]^ We identified the FOXO3/IRF4 axis as a transcriptional regulator of lipid metabolism. IRF4 is known to be important for plasma cell survival and differentiation ^[^ ^62^ ^]^ and is also implicated in lymphoid malignancy development.^[^ ^63^ ^]^ In B cells, loss of IRF4 is correlated with mitochondria elimination and disrupted lipid metabolism.^[^ ^64^ ^]^ While IRF4 regulates lipolysis in adipocytes,^[^ ^26^, ^36^ ^]^ a study on its role in tumor metabolism is still under investigation. Our findings of the decreased IRF4 due to CAMK2D suppression are consistent with tumor and lipid phenotypes of IRF4.

In summary, our study indicates that CAMKIIδ is a molecular target for the treatment of BCL. Furthermore, our results raise the possibility of targeting CAMKIIδ to treat other malignancies with FOXO3A‐induced lipid dysfunction.

## 4 Experimental Section

### Transgenic Mice

All animal procedures were approved by the IACUC of the City of Hope Medical Center at Duarte, CA, and Zhejiang University. The Breeding was set up between *Eµ‐Myc* mice (The Jackson Laboratory, stock no. 0 02728) and *Camk2d^‐/‐^* mice (generated by COH transgenic animal core) to generate *Eµ‐Myc: Camk2d^‐/‐^* and *Eµ‐Myc*: *Camk2d^wt/wt^* littermate mice. The mice were monitored for lymphoma development and humanely sacrificed at the terminal. Tumors were collected as required for further analysis.

The c‐MYC (GenBank accession number: NM_01 0849.4) and BCL2 (GenBank accession number: NM_0 09741.5) knockin floxed mice were generated with CRISPR/Cas‐mediated genome engineering by Cyagen Biosciences (Suzhou) Inc. In brief, the “mouse Myc CDS‐P2A‐Bcl2 CDS‐rBG polyA” cassette was cloned into Hipp11 (H11) locus, and a CAG promoter‐loxP‐PGK‐Neo‐6*SV40 polyA‐loxP was placed upstream of the cassette such that the expression of MycP2A‐Bcl2 cassette will be dependent on the expression of Cre recombination. Cas9 mRNA and gRNA were co‐injected into fertilized eggs with donor vector to generate targeted conditional knockin offspring. Mice were maintained on a C57BL/6J background. The B6‐CD19‐Cre transgenic mice were purchased from Gempharmatech Co., Ltd, (stock no. T003785). Heterozygote Myc‐Bcl2^ki/wt^ mice were crossed with heterozygote CD19‐Cre^±^ mice to yield transgenic heterozygote CD19^±^, Myc‐BCL2^ki/wt^ (CD19^+^ ki/wt, DEL) and non‐transgenic CD19^‐/‐^, Myc‐BCL2^ki/wt^ (CD19^‐^ ki/wt, WT) littermates.

*Camk2d^flox/flox^* mice were generated by Zhejiang Chinese Medicine University transgenic animal core. To get conditional *Camk2d* knockout DEL and wildtype littermates, heterozygote *Camk2d^flox/wt^, CD19‐Cre^+/+^* mice were crossed with *Camk2d^flox/wt^, Myc‐Bcl2^+/+^* mice to yield *Camk2d^flox/flox^, CD19^+^ ki/wt* (*Camk2d^‐/‐^ DEL*) and *Camk2d^wt/wt^, CD19^+^ ki/wt* (*Camk2d^wt/wt^ DEL*) reared under the same conditions.

### Mouse Tumor Histopathology and IHC Staining

Tumor specimens were prepared and analyzed by ImageJ software. Tumors were fixed in 4% PBS‐buffered formalin; dehydrated and embedded in paraffin; and sectioned and processed for hematoxylin and eosin (H&E) staining and IHC staining. The staining was performed by the Pathology Core of the City of Hope Medical Center. The primary antibodies used were FOXO3A (75D8) Rabbit antibody (Cat#2497, CST, USA), CD45R (B220) Monoclonal Antibody (RA3‐6B2) (eBioscience, USA), Anti‐c‐Myc (Y69) antibody (Cat#ab32072, Abcam, USA), PLIN3 Rabbit anti‐Human Polyclonal Antibody (Cat#LS‐B2539, LSBio, USA). Images were captured by a Leica DM 4000B microscope (Solms, Germany).

### Tissue Microarrays and IHC

A tissue array chip containing benign lymph nodes and DLBCL lymphoid tissues was obtained from Shanghai Outdo Biotech. Each chip comprised 85 tissue dots (77 with DLBCL and 8 benign lymph nodes from patients with tonsillitis). The EnVision + detection system (Dako) was used per the manufacturer's instructions. The study protocol was approved by the Shanghai Qutdo Biotech Company Ethics Committee.

### Cells and Reagents

Burkitt's lymphoma cell lines (Namalwa, Rael, Mutu) and double‐hit lymphoma cell line (SU‐DHL‐6, SU‐DHL‐4) were kindly provided by Dr. John Chan's lab from the Department of Pathology of the City of Hope. Cells were cultured with RPMI 1640 (Thermo Fisher Scientific, USA) + 10%FBS + P/S at 37 °C in a 95% air, 5% CO2 humidified incubator. TET was dissolved in stock solutions with dimethyl sulfoxide (DMSO; Santa Cruz, USA) at 10 mM for in vitro cell treatment. TET was dissolved in 1 × PBS for in vivo oral gavage.

### CAMK2D Knockout by the CRISPR/Cas9 System

The sgRNA sequences targeting human CAMK2D were designed using the CRISPR online design tool (www.genome‐engineering.org/crispr). The designed sequence was cloned into the pX458 plasmid (Addgene). The 20 nt guiding sequences targeting the exon of human CAMK2D are shown below: 5′‐CACCGGACGAGTATCAGCTTTTCG and 5′‐AAACCGAAAAGCTGATACTCGTCC. The sgRNA‐containing pX458 vector was electroporated into cells with the Cell Line Nucleofector Kit V (Lonza, USA) following the manufacturer's protocol. After 48 h, green fluorescent protein‐positive (GFP+) cells were single‐cell sorted by fluorescence‐activated cell sorting (FACS) into 96‐well plates. Single clones were then expanded and screened by western blot analysis.

### Survival/Proliferation Assay

The MTS assay, which measures cell survival, was conducted with the CellTiter 96 Aqueous Cell Proliferation Kit (Promega). The IC_50_ was defined as the drug concentration that induced a 50% viability decrease.

### Survival Analysis

R package Survminer was used for survival analysis. A median of CAMK2D or CAMK2G expression level across all the samples was set as the cutoff. For those cases with both primary and recurred samples, the expression value in recurred samples was kept in survival analysis.

### Transmission Electron Microscope Imaging

Transmission electron microscope (TEM) was performed by electron microscope core at the City of Hope. For cell preparation to do TEM, the cell pellets were fixed with 2% glutaraldehyde in 0.1 M Cacodylate buffer (Na (CH3)2AsO2 ·3H2O), pH 7.2, at 4 °C, overnight. The cell pellets were washed three times with 0.1 M Cacodylate buffer, pH 7.2, post‐fixed with 1% OsO4 in 0.1 M Cacodylate buffer for 30 min and washed three times with 0.1 M Cacodylate buffer. The samples were then dehydrated through 60%, 70%, 80%, 95% ethanol, 100% absolute ethanol (twice), and propylene oxide (twice), and were left in propylene oxide/Eponate (1:1) overnight at room temperature. The vials were sealed. The next day the vials were left open for 2–3 h to evaporate the propylene oxide. The samples were infiltrated with 100% Eponate and polymerized at ≈64 °C for 48 h. Ultra‐thin sections (≈70 nm thick) were cut using a Leica Ultra cut UCT ultramicrotome with a diamond knife, picked up on 200 mesh copper EM grids. Grids were stained with 2% uranyl acetate for 10 min, followed by Reynold's lead citrate staining for 1 minute. Electron microscopy was done on an FEI Tecnai 12 transmission electron microscope equipped with a Gatan OneView CMOS camera. Mitochondria morphology was characterized based on their cristae structure.^[^ ^65^ ^]^

### Detection of Lipid Droplets by Microscopy Analysis

Lipid droplets were stained using the Bodipy 493/503 probe. After cell plating on glass coverslips and fixation, cells were washed and incubated with 1 µg mL^−1^ of Bodipy 493/503 diluted in 150 mM NaCl for 15 min at room temperature. Cells were then washed, and slides mounted with ProLongTM Gold antifade medium with DAPI (Invitrogen). The number and the area of Bodipy dots per cell of at least 100 cells per independent experiment were determined with Image J software.

### Flow Cytometry (FACS) Analysis

Flow cytometry experiments were performed on the BD Fortessa cytometer instrument. Cellular lipid content was measured by flow cytometry using the Bodipy 493/503 probe. Briefly, cells were washed, incubated with Bodipy (0.5 µg mL^−1^) for 15 min at 37 °C, then washed and resuspended with 1 × PBS with DAPI to exclude dead cells. Data were analyzed with FlowJo v10 software (Tree Star Inc., Ashland, OR, USA).

### Lipidomic Analysis

The lipidomic was performed by the University of Wisconsin Biotechnology Center. The lipid extraction protocol is based extensively on the method of Matyash.^[^ ^66^ ^]^ Lipids are extracted in a solution of 250 µL phosphate‐buffered saline (PBS), 225 µL methanol containing internal standards (Avanti SPLASH LipidoMix (cat. # 330707‐1EA) at 10 µL per sample) and 750 µL methyl tert‐butyl ether (MTBE). Samples were analyzed by UHPLC/MS and UHPLC/MS/MS in positive ion and negative ion modes. Assignment of lipid identities to mass and retention time signal pairs was made using Lipid Annotator software (Agilent) ^[^ ^67^ ^]^ and the LC/MS/MS data. This database is then used by Profinder software (Agilent) to align sample retention times and extract and integrate ion chromatograms for each lipid in each sample LC/MS data file. These integrations are then reviewed for accuracy and a comma‐separated value file is exported for further analysis.

### Extracellular Flux Assays

Extracellular oxygen consumption rate (OCR) and extracellular acidification rate (ECAR) were determined with a Seahorse XFe24 Extracellular Flux Analyzer according to the manufacturer's instructions. All materials and compounds were obtained from Agilent Technologies. The day before the assay, the sensor cartridge was placed into the calibration buffer medium to hydrate overnight. Seahorse XF24 microplate wells were coated with 25 µL of Cell‐Tak (Corning;354 240) solution at a concentration of 22.4 µg mL^−1^ and kept at 4 °C overnight. On the day of assay, 2.5 × 10^5^ cells per well were plated in an XF24 Cell Culture Microplate coated with Cell‐Tak. Then the cells were incubated in XF RPMI medium (without phenol red) supplemented with 2 mM glutamine, 10 mM glucose, and 1 mM pyruvate for 45 min at 37 °C (non‐CO_2_ incubator) before the assay.

For Seahorse XF Cell Mito Stress Test Kit (Agilent Technologies) Initially, baseline cellular OCR is measured, from which basal respiration can be derived by subtracting non‐mitochondrial respiration. Next oligomycin (1.5 µM), a complex V inhibitor, is added and the resulting OCR is used to derive ATP‐linked respiration (by subtracting the oligomycin rate from baseline cellular OCR) and proton leak respiration (by subtracting non‐mitochondrial respiration from the oligomycin rate). Next carbonyl cyanide‐p‐trifluoromethoxyphenyl‐hydrazon (FCCP) (0.5 µM), a protonophore, is added to collapse the inner membrane gradient, allowing the ETC to function at its maximal rate, and maximal respiratory capacity is derived by subtracting non‐mitochondrial respiration from the FCCP rate. Lastly, Rotenone/Antimycin (0.5 µM), inhibitors of complex III and I, are added to shut down ETC function, revealing the non‐mitochondrial respiration.

To determine the part of the OCR dependent on FAO, the final concentration of 3 µM Etomoxir, 2 µM UK5099, or 3 µM BPTES was added. Figures were graphed by Prism GraphPad.

For Seahorse XF Glycolytic Rate Assay, ECAR and OCR were measured at the basal stage (basal glycolysis + mitochondrial acidification), in response to Rot/AA (inhibitors of mitochondrial electron transport chain; compensatory glycolysis) and 2‐deoxy‐D‐glucose (a glucose analog; post‐2‐DG acidification). The basal and compensatory glycolytic rates were calculated using the Seahorse Glycolytic Rate Assay Report Generator, and account for the contribution of CO_2_ to extracellular acidification derived from mitochondrial respiration.

### Proteomic Analysis by LC‐MS/MS—Protein Extraction

The sample was sonicated three times on ice using a high‐intensity ultrasonic processor (Scientz) in lysis buffer (8 M urea, 1% protease inhibitor cocktail). (Note: For PTM experiments, inhibitors were also added to the lysis buffer, e.g., 3 µM TSA and 50 mM NAM for acetylation, 1% phosphatase inhibitor for phosphorylation). The remaining debris was removed by centrifugation at 12 000 g at 4 °C for 10 min. Finally, the supernatant was collected, and the protein concentration was determined with a BCA kit according to the manufacturer's instructions.

Proteomic Analysis by LC‐MS/MS—Trypsin Digestion

The sample was slowly added to the final concentration of 20% v/v TCA to precipitate protein, then vortexed to mix and incubated for 2 h at 4 °C. The precipitate was collected by centrifugation at 4500 g for 5 min at 4 °C. The precipitated protein was washed with pre‐cooled acetone three times and dried for 2 h. The protein sample was then redissolved in 100 mM TEAB and ultrasonically dispersed. Trypsin was added at a 1:50 trypsin‐to‐protein mass ratio for the first digestion overnight. The sample was reduced with 5 mM dithiothreitol for 60 min at 37 °C and alkylated with 11 mM iodoacetamide for 45 min at room temperature in darkness. Finally, the peptides were desalted by the C18 SPE column.

Proteomic Analysis by LC‐MS/MS—Affinity Enrichment

Peptide mixtures were first incubated with IMAC microspheres suspension with vibration in loading buffer (50% acetonitrile/0.5% acetic acid). To remove the non‐specifically adsorbed peptides, the IMAC microspheres were washed with 50% acetonitrile/0.5% acetic acid and 30% acetonitrile/0.1% trifluoroacetic acid, sequentially. To elute the enriched phosphopeptides, the elution buffer containing 10% NH_4_OH was added and the enriched phosphopeptides were eluted with vibration. The supernatant containing phosphopeptides was collected and lyophilized for LC‐MS/MS analysis.

Proteomic Analysis by LC‐MS/MS—LC‐MS/MS Analysis

The tryptic peptides were dissolved in solvent A (0.1% formic acid, 2% acetonitrile/in water), and directly loaded onto a homemade reversed‐phase analytical column (25‐cm length, 75/100 µm i.d.). Peptides were separated with a gradient from 6% to 24% solvent B (0.1% formic acid in acetonitrile) over 70 min, 24% to 35% in 14 min, and climbing to 80% in 3 min then holding at 80% for the last 3 min, all at a constant flow rate of 300 nL min^−1^ on a nanoElute UHPLC system (Bruker Daltonics). The peptides were subjected to a capillary source followed by the timsTOF Pro (Bruker Daltonics) mass spectrometry. The electrospray voltage applied was 1.60 kV. Precursors and fragments were analyzed at the TOF detector, with an MS/MS scan range from 100 to 1700 m/z. The timsTOF Pro was operated in parallel accumulation serial fragmentation (PASEF) mode. Precursors with charge states 0 to 5 were selected for fragmentation, and 10 PASEF‐MS/MS scans were acquired per cycle. The dynamic exclusion was set to 30 s.

Proteomic Analysis by LC‐MS/MS—Database Search

The resulting MS/MS data were processed using the MaxQuant search engine (v1.6.6.0). Tandem mass spectra were searched against the human SwissProt database Homo_sapiens_9606_SP_20 191 115 (20 380 entries) concatenated with the reverse decoy database. Trypsin/P was specified as a cleavage enzyme allowing up to 2 missing cleavages. The mass tolerance for precursor ions was set as 20 ppm in the first search and 20 ppm in the main search, and the mass tolerance for fragment ions was set as 0.02 Da. Carbamidomethyl on Cys was specified as fixed modification, and acetylation on protein N‐terminal and oxidation on Met, acetylation on Lys/ phosphorylation on Ser, Thr, and Tyr were specified as variable modifications. FDR was adjusted to < 1%.

### Lentivirus Preparation and Infection

pLKO.1‐shFOXO3A and pLKO.1‐shScramble were packaged with pMD2.G and psPAX2. Plasmids were co‐transfected into 293T cells in the 6‐well plate. The lentiviral particles were harvested at 48 and 72 h post‐transfection. To infect lymphoma cells, the lentiviruses were added into cells with the existence of 4 µg mL^−1^ polybrene (H9268, Sigma–Aldrich). The leukemia cells were further subject to “spinoculation” at 1000 rpm, 32 °C for 90 min. The infected cells were selected with puromycin (P8833, Sigma–Aldrich).

### Chromatin Immunoprecipitation Assay

The ChIP assay was performed using the Chromatin IP Kit purchased from Merck (cat. no. 17–295). Briefly, cells were crosslinked with 1% formaldehyde in 10 cm culture dishes for 10 min at 37 °C. Then, cells were washed, and cell pellets were resuspended in 200 µL SDS Lysis Buffer. After being fragmented via Bioruptor sonication, the chromatin was incubated with anti‐FOXO3A antibody or IgG overnight at 4 °C with rotation. Then, the ChIP‐Grade Protein A beads were added to each immunoprecipitation reaction and incubated for another 1 h at 4 °C with rotation. The protein A agarose/antibody/histone complex was then washed and de‐crosslinked using 5 M NaCl and Proteinase K for 2 h at 65 °C. Finally, DNA was purified by phenol/chloroform extraction and ethanol precipitation and subjected to downstream assays (ChIP‐qPCR). All primer sequences used for ChIP‐qPCR are listed in Supplementary Data.

### Immunoprecipitation Assay

Immunoprecipitation was done using the ANTI‐FLAG M2 Magnetic Beads (M8826, Sigma, St. Louis, MO). After treatment, cell protein was extracted with IP lysis buffer (Thermo, Waltham, MA). The cell lysate was then incubated with pre‐washed anti‐FLAG M2 beads (50 µL) overnight at 4 °C with gentle rotation. Immunoprecipitated complexes were collected by magnetic separator and washed three times with 1 mL washing buffer by re‐suspension and magnetic separation. The immunoprecipitant was eluted by elution buffer and detected by Western blot analysis.

### In Vitro Kinase Assay

Human recombinant CAMKIIδ (Cat#ab84552, Abcam, USA) or CAMKIIγ (Cat#ab132986, Abcam, USA) protein was pre‐activated in a reaction containing calcium, calmodulin, and ATP 10 min at 30 °C. 3 x FLAG FOXO3A wild type or mutants were pulled down by anti‐FLAG M2 beads (50 µl) overnight and added into kinase reaction for 5 min. Reaction was quenched with lane marker sample buffer and phosphorylation was examined using western blot analysis.

### Cellular Thermal Shift Assay

Cellular thermal shift assay was used to evaluate the interactions between TET and CAMKIIδ in cells following the protocol.^[^ ^39^ ^]^ First, Mutu cells were treated with DMSO or 10 µM TET for 1 h. Then the treated cells were collected into a 200 µL tube and heated for 3 min from 45 to 52.5 °C in a C1000 Touch Thermal Cycler (Bio‐Rad, USA). Subsequently, cell lysates were freeze‐thaw three times. Protein lysates were extracted and used for Western blot analysis.

### Xenograft Mouse Model

All animal procedures were approved by the Institutional Animal Care and Use Committee (IACUC) in the City of Hope. 8‐week NSG (NOD.Cg‐Prkdcscid Il2rgtm1Wjl/SzJ) mice were purchased from the Jackson Laboratory (Strain #0 05557). The animals were maintained under specific pathogen‐free conditions in alternating 12‐h light/dark cycles and a temperature‐controlled room with food and water ad libitum. 5 × 10^5^ H9 cells suspended in 1 × PBS were injected subcutaneously in the right flank of female NSG mice. After the xenografted tumors reached a volume of ≈100 mm^3^, mice were randomized to 2 groups: (1) PBS: 100 µL d^−1^ n = 8; (2) TET: 100 mg kg d^−1^ (100 µL) n = 8. TET was dissolved in 1 × PBS and then administrated via oral gavage once a day for 12 days. Tumors and body weight were measured three times a week with Vernier calipers, and the tumor volume (mm^3^) was calculated as length × width^2^/2. Mice were euthanized, and tumor tissues were collected on the 32nd day of inoculation for further analysis.

### Cycloheximide (CHX) Chase Assay

Cells were treated with 50 mM cycloheximide (CHX) and harvested at the indicated time points. Protein was extracted from the cells and subjected to western blot analysis. Protein levels were measured with the densitometric intensity.

### Western Blot Analysis

Cell specimens were washed twice with PBS buffer; total cellular protein was extracted using Radio‐Immunoprecipitation Assay buffer (RIPA). Cell extracts were subjected to sodium dodecyl sulfate‐polyacrylamide gel electrophoresis (SDS‐PAGE; 10% polyacrylamide gels); then they were transferred to polyvinylidene difluoride (PVDF) membranes (Bio‐Rad) and blocked with 5% nonfat milk (Bio‐Rad) in TBS‐Tween 20 (TBST). The membranes were then reacted with primary antibodies overnight at 4 °C. After 3 washes with TBST, membranes were probed with a horseradish peroxidase‐conjugated secondary antibody for 1 h at room temperature and reacted with SuperSignal West Pico Chemiluminescent Substrate (Thermo). The following antibodies used in this work were from Abcam: Anti‐c‐Myc antibody [Y69] (#ab32072), Anti‐c‐Myc (phospho S62) antibody (#ab51156), Anti‐c‐Myc (phospho T58) antibody (#ab85380), Anti‐CamKII gamma antibody [8G10C1] (# ab201966), Anti‐CaMKII delta antibody [EPR13095] (#ab181052). The following antibodies used in this work were from Cell Signaling Technology: FoxO3a (D7D3Y) Mouse monoclonal antibody (#99 199), GAPDH (14C10) Rabbit antibody (#2118S), HSP90 (C45G5) Rabbit antibody (#4877S). ANTI‐FLAG M2 mouse monoclonal antibody was purchased from Sigma (#F1804). Phosphoserine/threonine Mouse antibody (Unlabeled, Clone: 22A) was purchased from BD (#612 549). USP7 recombinant rabbit monoclonal antibody [JB80‐36] was purchased from HuaBio (# ET7107‐53).

### Statistical Analysis

Student's t‐test (two‐sided) was applied to compare the two groups of samples, and changes were considered statistically significant for p < 0.05. For intercomparison of more than 2 groups, a one‐way ANOVA followed by a post‐hoc test was used. In the Figures, changes are noted using *p < 0.05 and **p < 0.01. The data were normally distributed and variation within and between groups was not estimated. The sample size was not pre‐selected, and no inclusion/exclusion criteria were used. Survival in mouse experiments was represented with Kaplan–Meier curves, and significance was estimated with the log‐rank test (Prism GraphPad). The data shown in the bar graphs are the mean and standard deviation (s.d.) of at least three biological replicates. Statistical analysis was conducted using the Microsoft Excel or GraphPad Prism software packages.

## Conflict of Interest

The authors declare no conflict of interest.

## Author Contributions

J.Z., S.X., H.F., and D.W. contributed equally to this work. Z., S.X., H.F., and D.W. performed animal and cell experiments. C.O. and J.Y. performed RNA‐seq analysis. Y.S., Z.H., M.Z., Y.Z., J. Z., S.Z., and Y.G. provided technical support. X.L., Y.L., M.T., and Y.W. helped with data analysis. L.L., W.C., D.H., and M.F. provided conceptual and technical advice and overall support. W.H. and Y.G. supervised the project.

## Supporting information



Supporting Information

## Data Availability

The data that support the findings of this study are available on request from the corresponding author. The data are not publicly available due to privacy or ethical restrictions.
