# Supplementary material for: CAMKIIδ Reinforces Lipid Metabolism and Promotes the Development of B Cell Lymphoma
Source: Adv Sci (Weinh). 2025 Jan 22;12(10):2409513. doi: 10.1002/advs.202409513 (PMC11905072; doi:10.1002/advs.202409513)
Supplement: Supplementary file 1 — Supporting Information [file ADVS-12-2409513-s001.pdf]

ADVANCED SCIENCE
Open Access

Supporting Information

for *Adv. Sci.*, DOI 10.1002/advs.202409513

CAMKIIδ Reinforces Lipid Metabolism and Promotes the Development of B Cell Lymphoma

Jiawei Zhang, Senlin Xu, Hui Fang, Dehao Wu, Ching Ouyang, Yunfei Shi, Zhenkang Hu, Mingfeng Zhang, Yaoyao Zhong, Junwei Zhao, Yichao Gan, Shize Zhang, Xiaoqian Liu, Jie Yin, Yuan Li, Mengyue Tang, Yingda Wang, Ling Li, Wing C Chan, David Horne, Mingye Feng, Wendong Huang* and Ying Gu**

Figure. S1

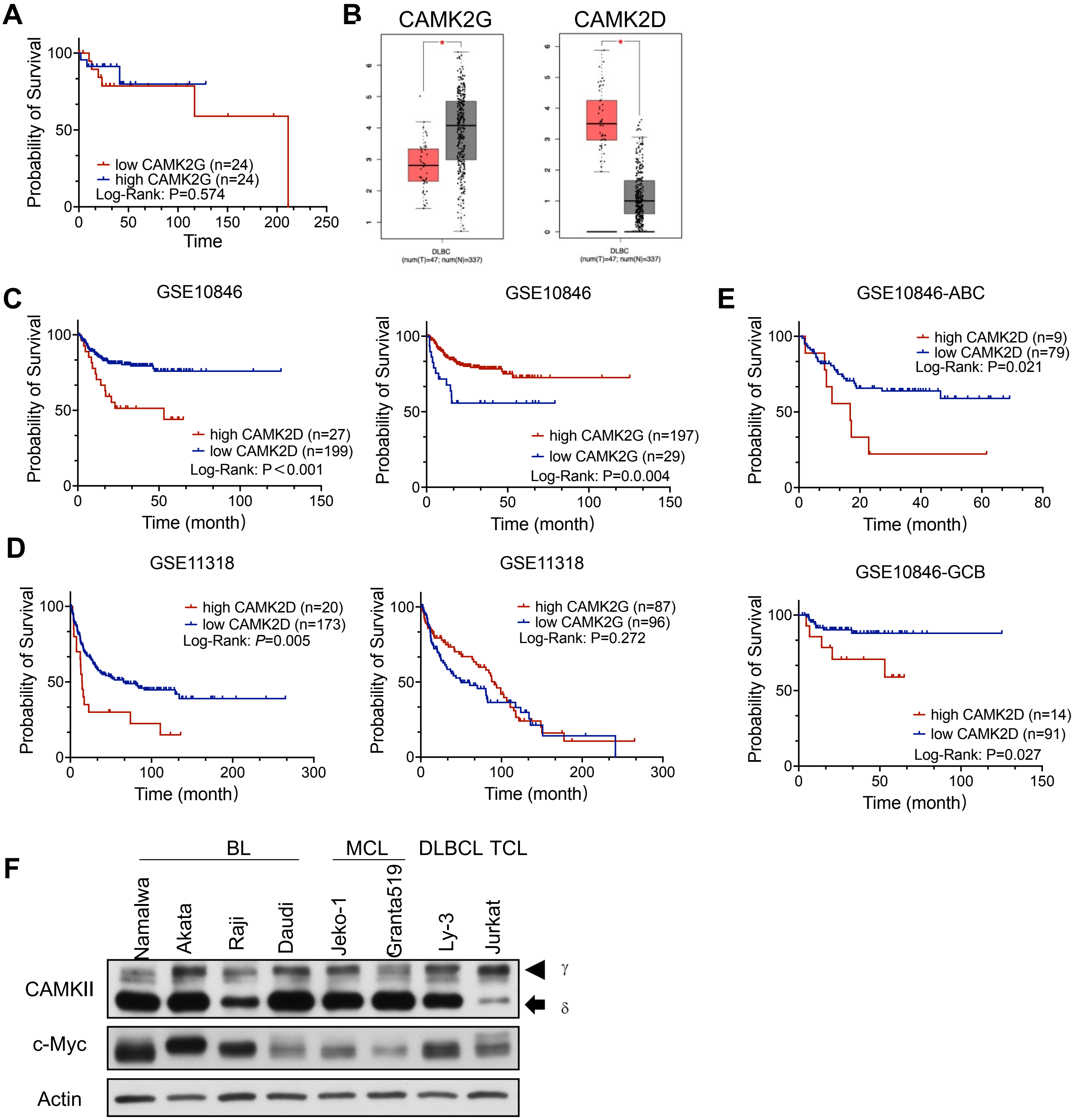

Figure. S1. (Related to Figure. 1) CAMKIIδ is critical for BCL proliferation. (A) Kaplan-Meier survival of TCGA-DLBCL patients with either high (n=24) or low (n=24) CAMK2G expression. **(B)** Normalized expression level (TPM) of CAMK2G (left) and CAMK2D (right) relative to GAPDH in TCGA-DLBCL cohort (n=47) compared to normal cohort (n=337). *p<0.05. **(C)** Kaplan-Meier survival analysis of patients from the GSE10846 DLBCL dataset with high or low CAMK2D and CAMK2G expression. P values were calculated using the log-rank test. **(D)** Kaplan-Meier survival analysis of patients from the GSE11318 DLBCL dataset with high or low CAMK2D and CAMK2G expression. P values were calculated using the log-rank test. **(E)** Kaplan-Meier survival of CAMK2D expression in patients from the GSE10846 dataset with subtype information. P values were calculated using the log-rank test. **(F)** Representative western blot results of indicated protein expression from lymphoma cell lines. Data are represented of three independent experiments.

Figure. S2

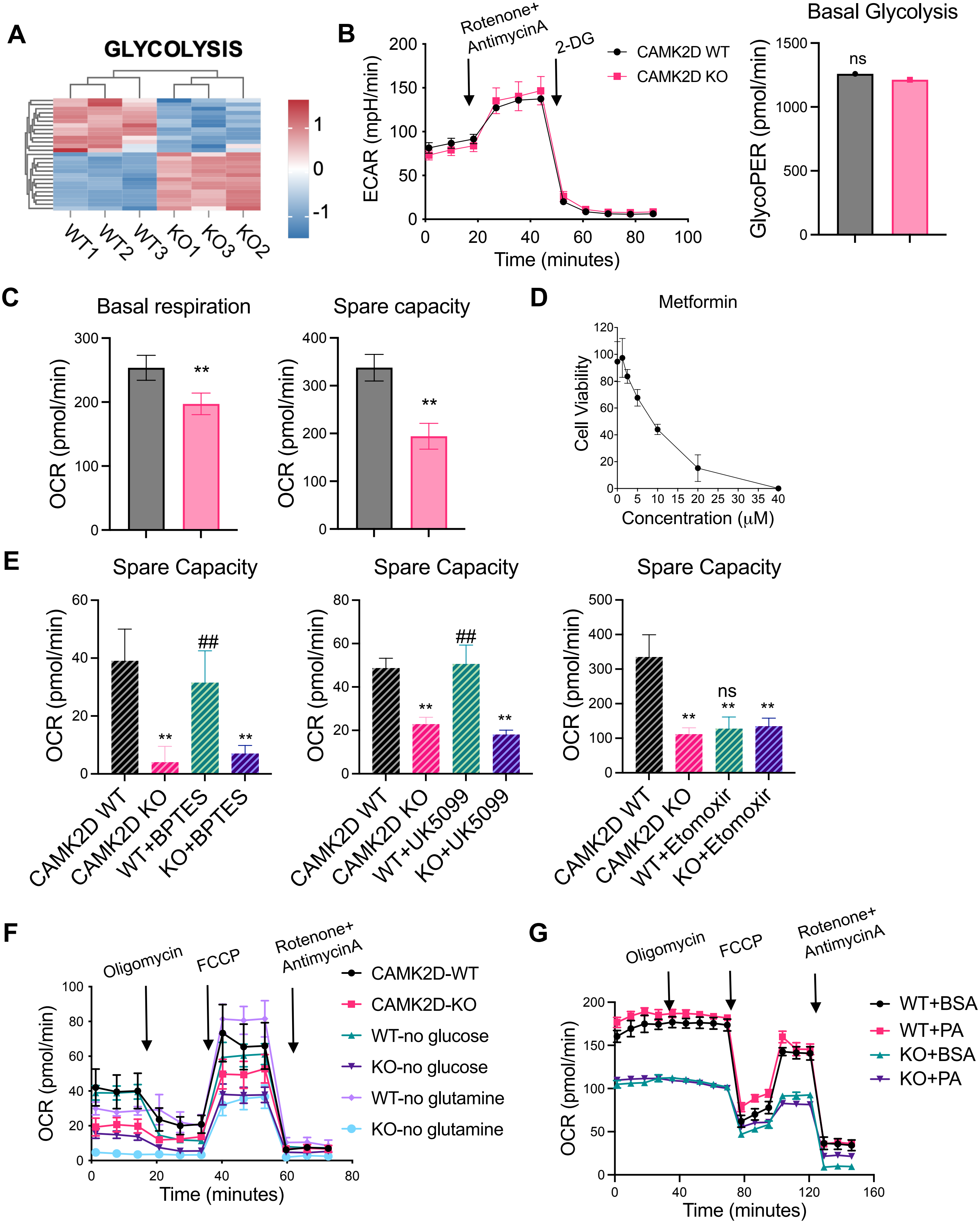

A
GLYCOLYSIS
WT1
WT2
WT3
KO1
KO3
KO2
1
0
-1
B
Rotenone+
AntimycinA
2-DG
CAMK2D WT
CAMK2D KO
ECAR (mpH/min)
Time (minutes)
Basal Glycolysis
ns
GlycoPER (pmol/min)
C
Basal respiration
Spare capacity
OCR (pmol/min)
**
D
Metformin
Cell Viability
Concentration (μM)
E
Spare Capacity
OCR (pmol/min)
##
ns
CAMK2D WT
CAMK2D KO
WT+BPTES
KO+BPTES
WT+UK5099
KO+UK5099
WT+Etomoxir
KO+Etomoxir
F
Oligomycin
FCCP
Rotenone+
AntimycinA
CAMK2D-WT
CAMK2D-KO
WT-no glucose
KO-no glucose
WT-no glutamine
KO-no glutamine
OCR (pmol/min)
Time (minutes)
G
Oligomycin
FCCP
Rotenone+
AntimycinA
WT+BSA
WT+PA
KO+BSA
KO+PA
OCR (pmol/min)
Time (minutes)

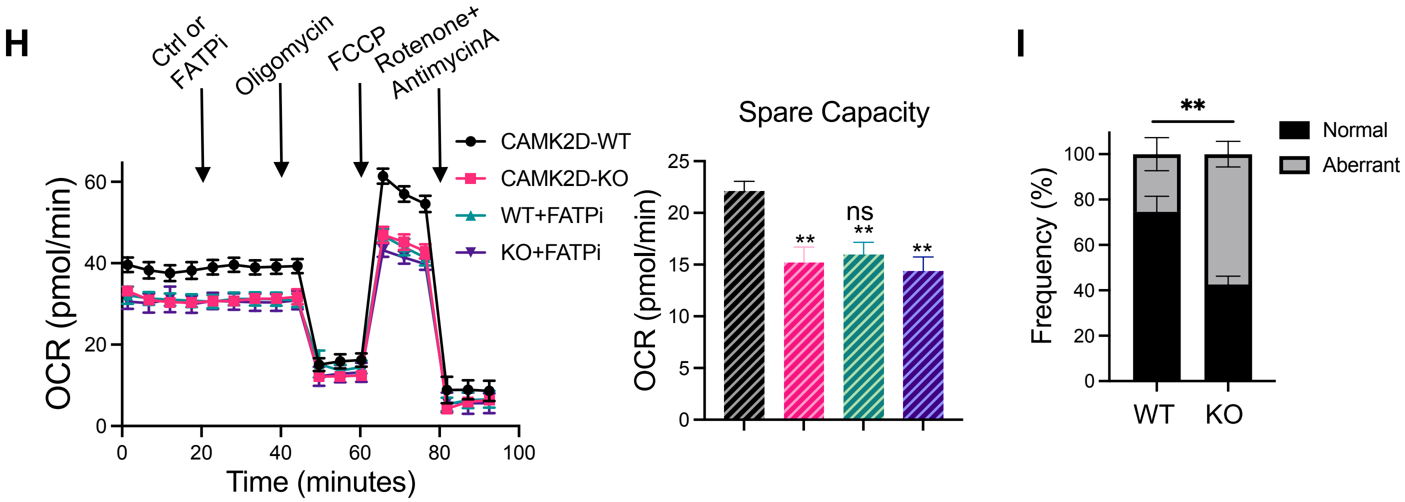

Figure. S2. (Related to Figure. 2) CAMK2D inhibition impaired mitochondria functions. (A) Heatmap analysis of selected genes from glycolysis pathway from bulk RNA-seq data between CAMK2D WT and KO cells. **(B)** Representative measures of extracellular acidification rate (ECAR) between Mutu CAMK2D WT and KO cells. Quantified basal glycolysis. ns: nonsignificant. The results are representative of three independent experiments. **(C)** Quantified basal respiration and spare capacity from Fig. 2B. **p<0.01. **(D)** Cell viability curve of Mutu cells treated with indicated dosages of metformin for 48 hours. Curve fits were obtained using Prism (GraphPad Software). The results are representative of three independent experiments. **(E)** Quantified spare capacity from Fig. 2D. **p<0.01 vs. CAMK2D WT. ##p<0.01 vs CAMK2D KO. **(F)** Representative measures of oxygen consumption rate (OCR) in cells with or without glucose or glutamine in the assay medium. **(G)** Representative measures of oxygen consumption rate (OCR) in cells supplied with palmitate or BSA control. The results are representative of independent experiments. **(H)** Representative measures of oxygen consumption rate (OCR) in cells treated with the FATP1 inhibitor. The results are representative independent experiments. **p<0.01 vs CAMK2D WT. n.s: nonsignificant vs. CAMK2D KO. **(I)** Quantified mitochondrial morphology from Fig. 2G. Mitochondria were characterized based on their cristae structure. Mitochondria were classified as normal if they showed classical lamellar cristae. All mitochondria that showed different cristae shapes or that were highly disordered were classified as aberrant. **p<0.01 vs CAMK2D WT.

Figure. S3

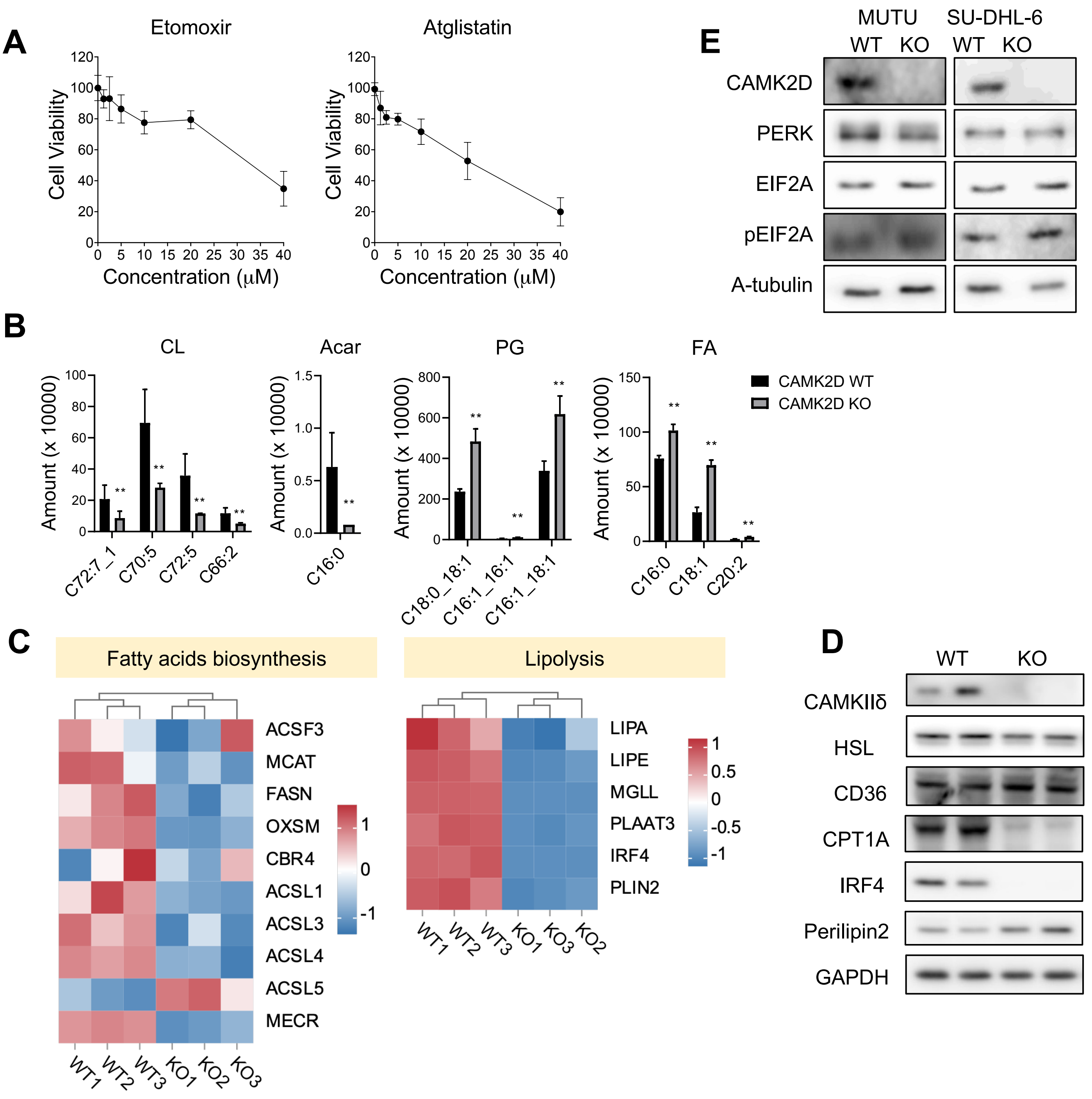

Figure. S3. (Related to Figure. 3) CAMKIIδ regulates lipid homeostasis in B cell lymphoma. (A) Cell viability curve of Mutu cells treated with indicated dosages of Etomoxir or Atglistatin for 48 hours. Curve fits were obtained using Prism (GraphPad Software). The results are representative of three independent experiments. **(B)** Bar chart of mitochondria-related lipids expression between CAMK2D WT and KO cells from lipidomic analysis. $^{**}p<0.01$. **(C)** Heatmap of selected gene expression from RNA-seq. **(D)** Representative western blot results of indicated protein expression related to lipid metabolism between CAMK2D WT and KO cells. The results are representative of three independent experiments. **(E)** Representative western blot results of the expression of indicated proteins in CAMK2D WT and KO cells. The results are representative of three independent experiments.

Figure. S4

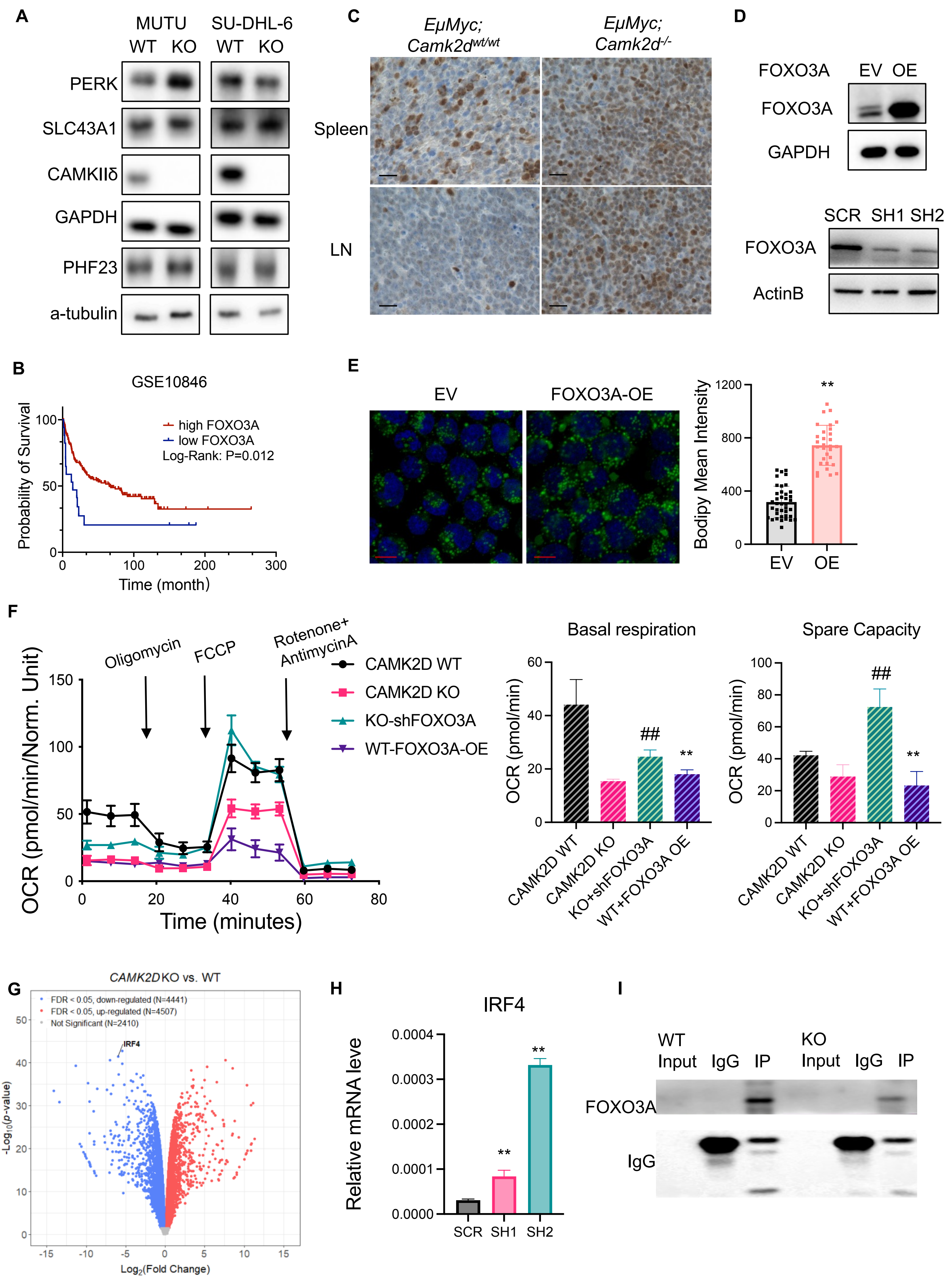

A
MUTU
SU-DHL-6
WT KO WT KO
PERK
SLC43A1
CAMKIIδ
GAPDH
PHF23
a-tubulin
C
EμMyc; Camk2d wt/wt
EμMyc; Camk2d -/-
Spleen
LN
D
FOXO3A
EV OE
FOXO3A
GAPDH
SCR SH1 SH2
FOXO3A
ActinB
B
GSE10846
high FOXO3A
low FOXO3A
Log-Rank: P=0.012
Probability of Survival
Time (month)
E
EV
FOXO3A-OE
Bodipy Mean Intensity
EV
OE
**
F
Oligomycin
FCCP
Rotenone+ AntimycinA
CAMK2D WT
CAMK2D KO
KO-shFOXO3A
WT-FOXO3A-OE
OCR (pmol/min/Norm. Unit)
Time (minutes)
Basal respiration
Spare Capacity
OCR (pmol/min)
CAMK2D WT
CAMK2D KO
KO+shFOXO3A
WT+FOXO3A OE
##
**
G
CAMK2D KO vs. WT
FDR < 0.05, down-regulated (N=4441)
FDR < 0.05, up-regulated (N=4507)
Not Significant (N=2410)
IRF4
-Log10(p-value)
Log2(Fold Change)
H
IRF4
Relative mRNA leve
SCR
SH1
SH2
**
I
WT Input
IgG
IP
KO Input
IgG
IP
FOXO3A
IgG

Figure. S4. (Related to Figure. 4) FOXO3A is a key substrate of CAMKIIδ. (A) Representative western blot results of indicated protein expression from indicated CAMK2D WT and KO cells. The results are representative of three independent experiments. **(B)** Kaplan-Meier survival of patients from the GSE10846 dataset with either high (n=157) or low (n=17) FOXO3A expression. P values were calculated using the log-rank test. **(C)** Magnified IHC staining image of FOXO3A from Figure 4D. The bar represents 10μm. **(D)** Representative western blot results showing protein expression in cells with FOXO3A knockdown or overexpression. The results are representative of three independent experiments. **(E)** Representative images of BODIPY 493/503 staining in cells with or without FOXO3A overexpression. EV: empty vector. **p<0.01 vs EV. The bar represents 10μm. **(F)** Representative measures of oxygen consumption rate (OCR) in cells with different FOXO3A levels. The results are representative of three independent experiments. **p<0.01 vs CAMK2D WT. $^{\#\#}$p<0.01 vs CAMK2D KO. **(G)** The volcano plot showed IRF4 expression from the RNA-seq experiment. **(H)** Relative IRF4 mRNA level from Mutu$^{CAMK2D-/-}$ cells transduced with scramble shRNA or shRNA target FOXO3A. **p<0.01 vs SCR. The results are representative of three independent experiments. **(I)** Immunoprecipitation fractions from FOXO3A-ChIP were analyzed by western blot and indicated protein expression was measured. The results are representative of three independent experiments.

Figure. S5

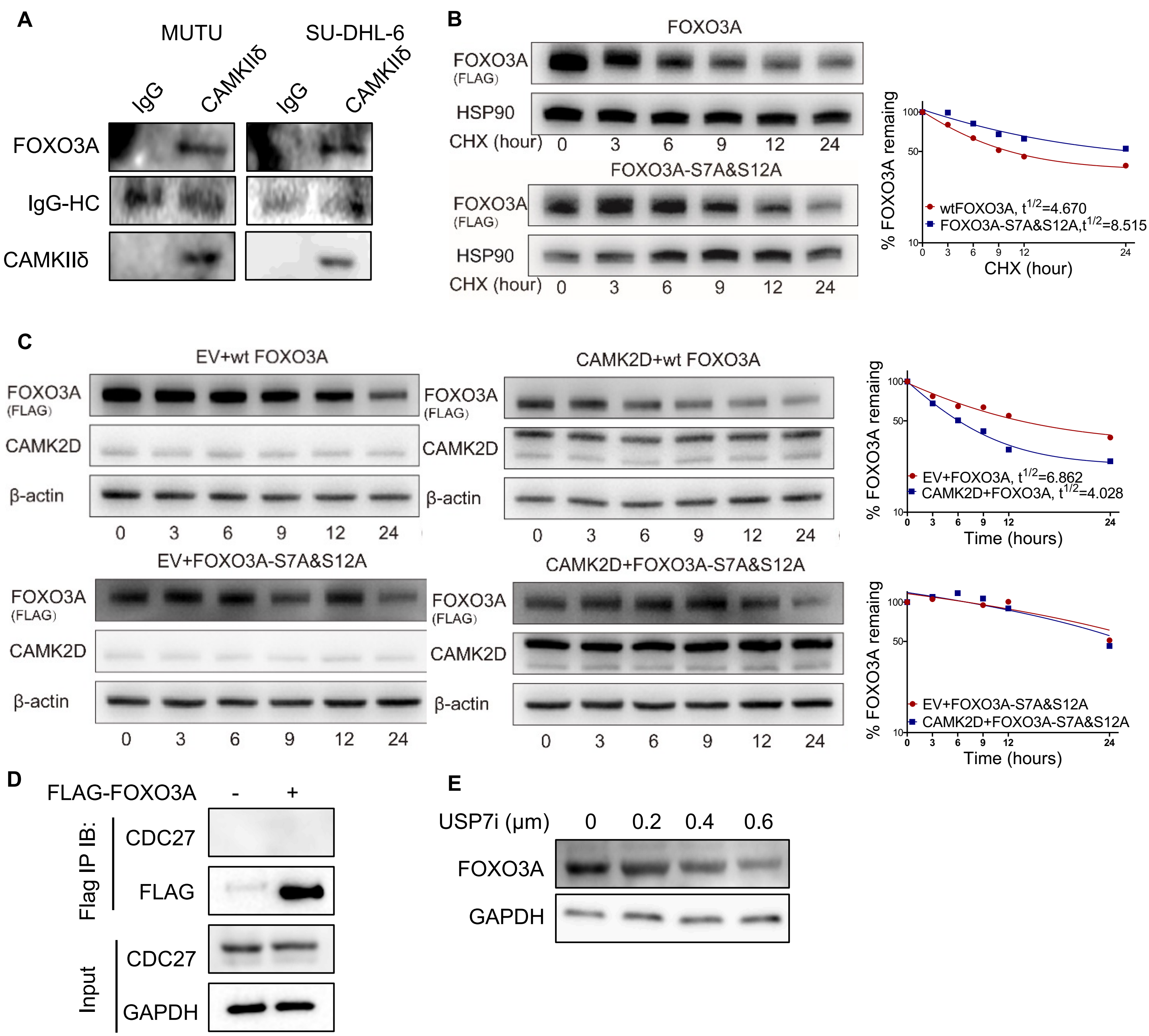

Figure. S5. (Related to Figure. 5) CAMKIIδ phosphorylates and destabilizes FOXO3A. (A) Endogenous co-immunoprecipitation performed in Mutu and SU-DHL-6 cells. Cell lysates were extracted and incubated with CAMKIIδ antibody or isotype IgG control, then precipitated with Protein A/G Magnetic Beads. The immunoprecipitant was analyzed by western blot using FOXO3A antibody for detection. **(B)** HEK293 cells expressed 3xFLAG-FOXO3A wild-type, and S7/12A were treated with CHX for the indicated times. The FOXO3A protein levels were analyzed by western blot with HSP90 as the loading control. Protein levels were measured with densitometric intensity. FOXO3A levels were quantified relative to HSP90 levels and graphed as the percentage of remaining FOXO3A protein after treatment. The results are representative of three independent experiments. **(C)** HEK293 cells co-transfected with the indicated plasmids were treated with CHX for the indicated times. The FOXO3A protein levels were then analyzed by western blotting. The results are representative of three independent experiments. **(D)** HEK293 cells transfected with 3xFLAG-FOXO3A were co-immunoprecipitated with FLAG beads; protein levels of CDC27 were analyzed by western blot. The results are representative of three independent experiments. **(E)** BCL cells overexpressed FOXO3A were treated with USP7 inhibitor at indicated concentrations for 24 h. The FOXO3A level was determined by western blot. The results are representative of three independent experiments.

Figure. S6

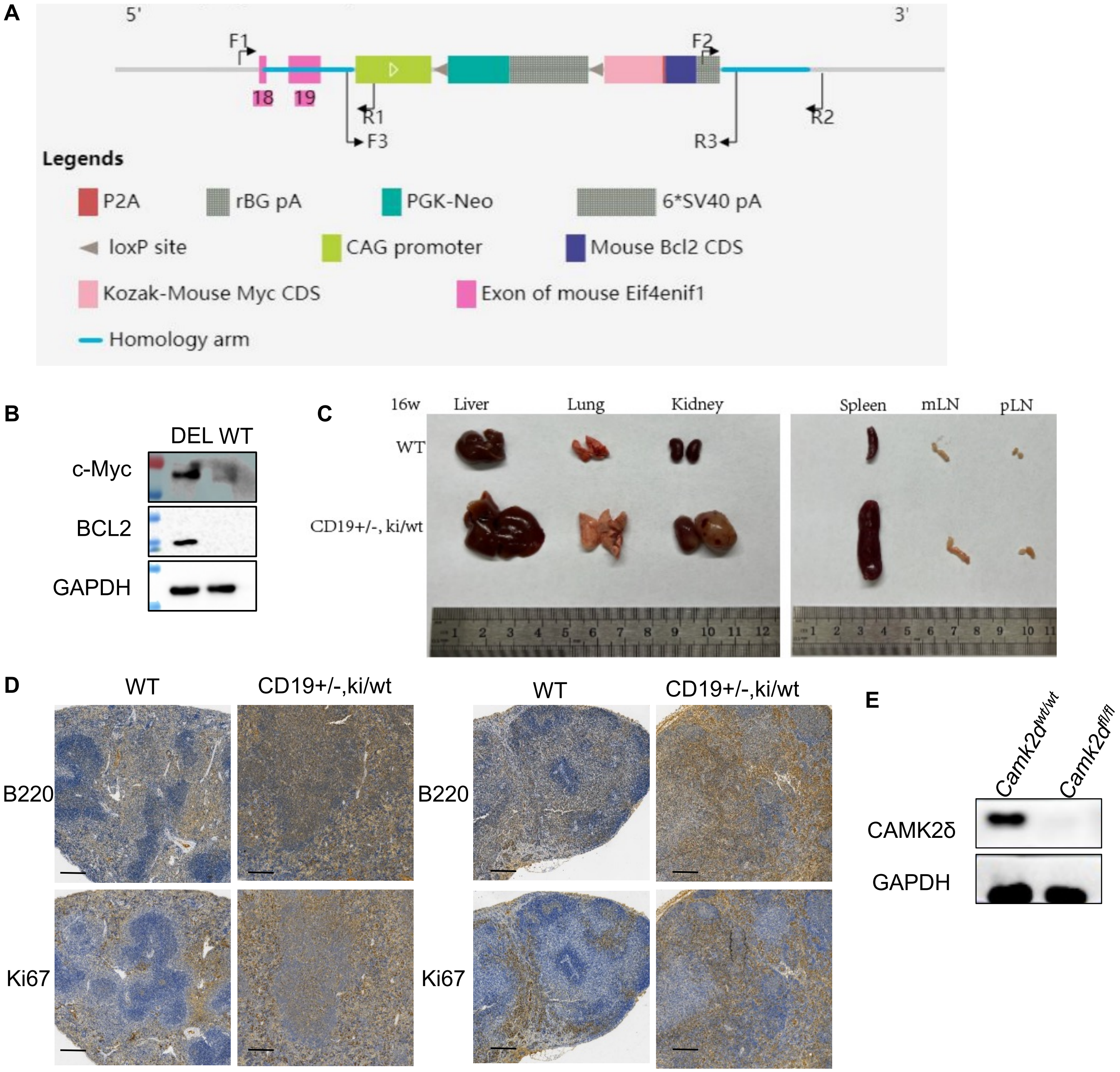

Figure. S6. (Related to Figure. 6) Generation and verification of c-Myc and BCL2 conditional co-expression mice (DEL). (A) Schematic diagram of the generation of c-MYC and BCL2 conditional Co-expression mice. **(B)** B cells were isolated from spleen of indicated mice and indicated proteins expression was detected by western blots. Data are representative of three independent experiments. **(C)** Representative anatomic images of liver, lung, kidney, spleen, and lymph nodes from wild type and DEL mice (16 weeks). **(D)** Representative images of IHC staining for B220 (CD45R) and Ki67 on spleens (left) and lymph node (right) sections. The bar represents 200μm. **(E)** B cells were isolated from spleen of CAMK2D wildtype or knockout DEL mice and CAMKIIδ protein expression was detected by western blots. Data are representative of three independent experiments..

Figure. S7

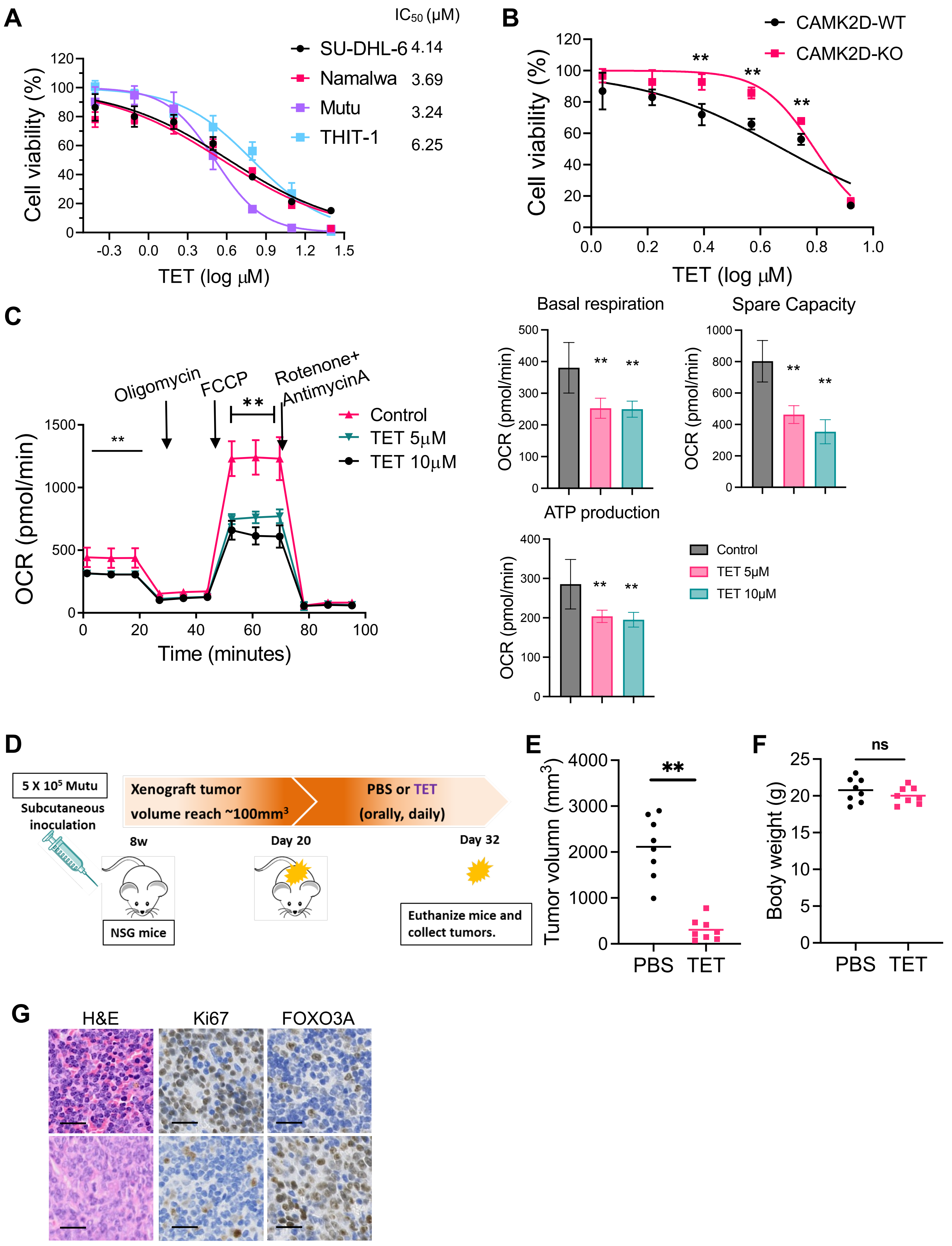
A
IC50 (μM)
SU-DHL-6 4.14
Namalwa 3.69
Mutu 3.24
THIT-1 6.25
Cell viability (%)
TET (log μM)
B
CAMK2D-WT
CAMK2D-KO
Cell viability (%)
TET (log μM)
C
Oligomycin
FCCP
Rotenone+ AntimycinA
Control
TET 5μM
TET 10μM
OCR (pmol/min)
Time (minutes)
Basal respiration
Spare Capacity
ATP production
OCR (pmol/min)
Control
TET 5μM
TET 10μM
D
5 X 10^5 Mutu
Subcutaneous inoculation
Xenograft tumor volume reach ~100mm³
PBS or TET (orally, daily)
8w
Day 20
Day 32
NSG mice
Euthanize mice and collect tumors.
E
Tumor volumn (mm³)
PBS
TET
F
ns
Body weight (g)
PBS
TET
G
H&E
Ki67
FOXO3A

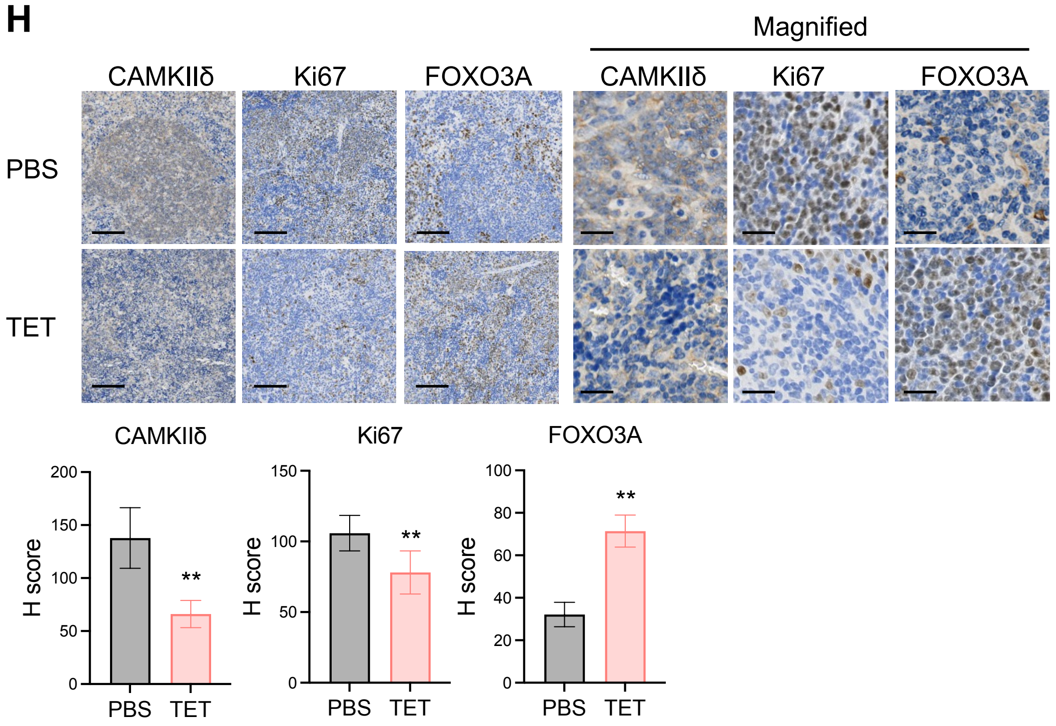

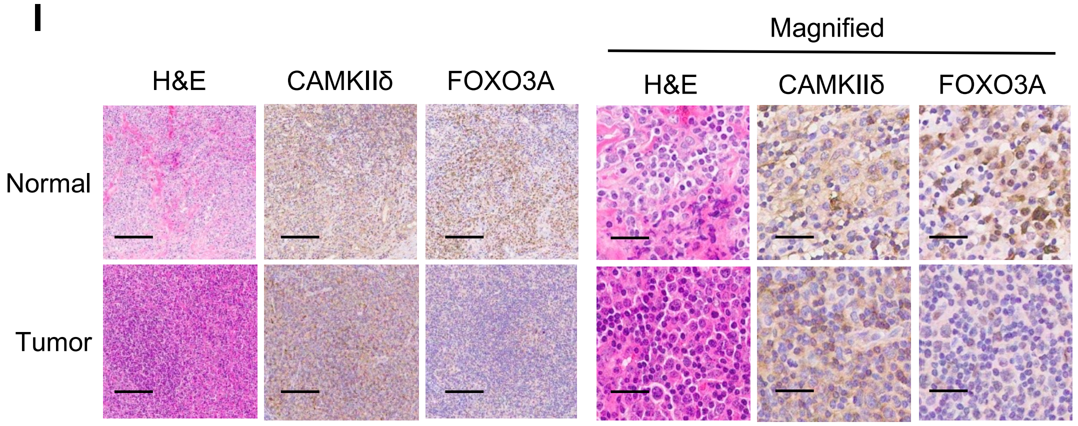

Figure. S7. (Related to Figure. 7) TET inhibits CAMKIIδ activity and B cell lymphoma proliferation *in vitro* and *in vivo*. (A) Cell viability curve of indicated B cell lymphoma cell lines treated with indicated dosages of TET for 24 hours. IC_{50} values and curve fits were obtained using Prism (GraphPad Software). The results are representative of three independent experiments. **(B)** Cell viability curve of SU-DHL-6 CAMK2D WT and KO cells treated with indicated dosages of TET for 24 hr. ** $p < 0.01$. The results are representative of three independent experiments. **(C)** Representative measures of oxygen consumption rate (OCR) after indicated dosages of TET treatment. **$p<0.01$. The results are representative of three independent experiments. **(D)** Scheme of Mutu-derived lymphoma in NSG mouse model, which was treated with TET for 12 days. **(E)** Tumor volume at the endpoint of the 32nd day. ** $p<0.01$ vs. PBS group. **(F)** Body weight of mice at the endpoint of the 32nd day in indicated groups. n.s. no statistical significance vs. the PBS group. **(G)** IHC staining images at higher magnification for Figure 7I. The bar represents 20µm. **(H)** Representative H&E and IHC images for CAMKIIδ, Ki67, and FOXO3A expression in DEL mice treated with either PBS (n=6) or TET (n=6). The bar represents 100µm and 20µm (magnified). ** $p<0.01$ vs. PBS group. **(I)** Representative H&E and IHC images for CAMKIIδ and FOXO3A expression in DLBCL tissue microarrays. The bar represents 50µm and 10µm (in magnified).
